# Middle Stone Age Bifacial Technology and Pressure Flaking at the MIS 3 Site of Toumboura III, Eastern Senegal

**DOI:** 10.1007/s10437-021-09463-5

**Published:** 2021-11-25

**Authors:** Viola C. Schmid, Katja Douze, Chantal Tribolo, Maria Lorenzo Martinez, Michel Rasse, Laurent Lespez, Brice Lebrun, David Hérisson, Matar Ndiaye, Eric Huysecom

**Affiliations:** 1 Laboratory of Archaeology and Population in Africa (APA), Anthropology Unit, Department of Genetics and Evolution, University of Geneva, Quai Ernest-Ansermet 30, 1205 Genève, Switzerland; 2 Research Institute on Archaeological Materials-Center of Research on Physics Applied to Archaeology (IRAMAT-CRP2A), CNRS-UMR 5060, University Bordeaux-Montaigne, Esplanade des Antilles, 33607 Pessac Cedex, France; 3 Inrap ARA, rue Louis Maggiorini 12, 69500 Bron, France; 4 Laboratory Archéorient, CNRS-UMR 5133, Maison de l’Orient et de la Méditerranée, University Lumière Lyon 2, 7 Rue Raulin, 69007 Lyon, France; 5 Laboratory of Physical Geography (LGP), CNRS-UMR 8591, Department of Geography, University Paris-Est Creteil, 1 place Aristide Briand, 92195 Meudon, France; 6 Anthropologie des Techniques, des Espaces et des Territoires au Pliocène et au Pléistocène (ArScAn-AnTET), CNRS-UMR 7041, MSH Mondes, Paris Nanterre University, 21 Allée de l’Université, 93023 Nanterre Cedex, France; 7 Laboratory of Prehistory and Protohistory, Institut Fondamental d’Afrique Noire, University of Cheikh Anta Diop de Dakar, 33 Route de la Corniche Ouest, Dakar, Senegal

## Abstract

Over the past decade, the increasing wealth of new archaeological data on the Middle Stone Age (MSA) in Senegal and Mali has broadened our understanding of West Africa’s contributions to cultural developments. Within the West African sequence, the phase of Marine Isotope Stage 3 (MIS 3, ca. 59-24 ka) yielded so far the best known and extensive archaeological information. The site of Toumboura III encompasses an occupation dated by optically stimulated luminescence (OSL) to between 40 ± 3 ka and 30 ± 3 ka. It provides the largest, well-dated, and stratified lithic assemblage in West Africa for the MSA and sheds light on an unprecedented cultural expression for this period, adding to the notable diversity of the late MSA in this region. We conducted a technological analysis of the lithic components following the *chaîne opératoire *approach. The lithic assemblage features a prevalence of bifacial technology and the exploitation of flakes as blanks for tool production. The craftspeople manufactured distinct types of bifacial tools, including small bifacial points shaped by pressure technique. The new data from Toumboura III demonstrate behavioral patterns that are entirely new in the region. By revealing behavioral innovations and technological particularities, these results on the techno-cultural dynamics during the MIS 3 phase of the MSA enhance our understanding of the complex Pleistocene population history in this part of Africa.

**Supplementary Information:**

The online version contains supplementary material available at 10.1007/s10437-021-09463-5.

## Résumé

Au cours de la dernière décennie, la multiplication des nouvelles données archéologiques sur le Middle Stone Age (MSA) au Sénégal et au Mali a mené à une meilleure introduction de l’Afrique de l’Ouest dans la discussion sur les développements culturels. Dans la séquence ouest-africaine, la phase relative au stade isotopique marin 3 (MIS 3, ~ 59 - 24 ka) est à ce jour la mieux documentée du point de vue archéologique. Le site de Toumboura III représente une occupation humaine datée entre 40 ± 3 ka et 30 ± 3 ka par luminescence stimulée optiquement (OSL). Il a fourni l’assemblage lithique stratifié MSA le plus abondant d’Afrique de l’Ouest et met en lumière une expression culturelle inédite pour cette période, ajoutant ainsi une industrie unique à la diversité considérable déjà reconnue pour le MSA tardif de cette région. Nous avons mené une analyse technologique des artefacts lithiques selon une approche des chaînes opératoires. L’assemblage lithique est caractérisé par une prévalence du façonnage bifacial et par l’exploitation d’éclats de façonnage comme supports d’outils. Les artisans ont confectionné différents types d’outils bifaciaux, dont des petites pointes bifaciales montrant la mise en oeuvre de la technique de façonnage par pression. Les nouvelles données obtenues à Toumboura III montrent des comportements jusque-là inconnus dans la région. Par la mise en évidence d’innovations comportementales et de particularités technologiques, ces résultats sur les dynamiques techno-culturelles au MSA au cours du MIS 3 contribuent à mieux comprendre l’histoire complexe du peuplement dans cette région.

## Introduction

Scientific efforts have intensified in the last decade to increase the knowledge about the West African Middle Stone Age (MSA) (e.g., Allsworth-Jones, 2019; Chevrier et al., 2016, 2018; Huysecom, 1987; Niang et al., 2018; Scerri et al., 2016, 2017, 2021). The accumulating archaeological data, especially from Senegal and Mali, reveal distinctive cultural developments that have enriched our understanding of human behavioral evolutionary history during this period. This archaeological record is of paramount importance in West Africa where paleoanthropological data from the Pleistocene record are generally very minimal (but see Harvati et al., 2011) and where demographic and biological scenarios take on meaning within the broader temporal scope of the continental-wide “African multi-regionalism” model (e.g., Scerri et al., 2018; Schlebusch & Jakobsson, 2018; Stringer, 2016).

The Marine Isotope Stage (MIS) 3 stands out as the best documented period within the West African MSA based on the occurrence of numerous sites (e.g., Allsworth-Jones, 2019; Chevrier et al., 2018; Tribolo et al., 2015). Previous works carried out at the Ounjougou site complex, Mali (e.g., Chevrier et al., 2018; Soriano et al., 2011), and the re-analysis and re-excavation of Tiémassas, Senegal (Niang et al., 2018, 2020), show great variability of industries during MIS 4 to MIS 3 in West Africa. Past human groups most frequently used discoid reduction, Levallois methods, bifacial shaping, and bipolar-on-anvil knapping of quartz cobbles (Chevrier et al., 2018; Soriano et al., 2011). Moreover, the presence of tanged pieces from the recently re-excavated stratified deposits and the old MSA collections at Tiémassas, as well as newly documented sites from the Lower Senegal Valley, suggest potential interregional cultural connections between the northern and western parts of Africa during the Late Pleistocene (Descamps, 1979; Niang et al., 2018; Scerri et al., 2016).

In contrast to the diversity of MSA industries represented at Ounjougou during MIS 4 to 3, the continuity of broadly homogeneous MSA traditions from ca. 65 to 25 ka at Tiémassas (Niang, et al., 2020) as well as the very young MSA dates of ca. 23–20 ka at Laminia, 11.6 ± 0.51 ka at Ndiayène Pendao, and 11.1 ± 0.58 ka at Saxomununya underpin an enduring existence of these Senegalese MSA sites into the onset of the Holocene (Scerri et al., 2017, 2021). These Senegalese assemblages echo unquestionably typical MSA features, such as Levallois and discoid reduction sequences, denticulates, notched pieces, side scrapers, and small foliate points (Scerri et al., 2021). Several other sites, extending from early MIS 3 into MIS 2 and even the onset of the Holocene, yielded evidence for the manufacture of bifacial tools. These sites include Kondo, Songona 1, Oumounaama Px, Kokolo 3, Dandoli Ouest, Draperies, and Oumounaama Butte in Mali, Birimi in Ghana, and Missira III in Senegal (Chevrier et al., 2018; Quickert et al., 2003; Scerri et al., 2021). In Mali, where the MIS 4/3 chrono-cultural framework is best known, there are sites with bifacial tools and those lacking bifacial pieces. Bifacial technology thus can be considered an appearing and disappearing techno-typological trait in the West African late MSA. This mosaic of the MSA documented in Mali, its stability and persistence in Senegal, and its contemporaneity with the Later Stone Age (LSA) industries, especially within Senegal (Chevrier et al., 2016, 2020) indicate cultural and spatial diversity.

Archaeological research in the Falémé Valley, eastern Senegal, has recently led to the documentation of the archaeological composition and the palaeoenvironment of the Ravin Blanc I site, a MSA site dated by OSL to ca. 125 ka. The MSA complex reveals evidence of Levallois and volumetric production and bifacial technology in the form of crudely shaped large bifacial pieces or bifaces (Douze et al., 2021). We studied the assemblage of the MIS 3 site of Toumboura III (hereafter, TMB III), also located in the Falémé Valley, which is currently the largest, well-dated, and stratified MSA site of the MIS 3 period in West Africa. The site was already known, in previous publications, for its bifacial component (Chevrier et al., 2016, 2018). However, our study of the lithic assemblage also emphasizes shaping within the general technical system and the use of pressure flaking, which is the only documented case so far in this region for the MSA. The unique technological signature of the lithic industry of TMB III adds to the MSA diversity already documented in Mali and other Senegalese sites.

## Presentation of the Site: Toumboura III

### Location and Excavation History

**Fig. 1:**
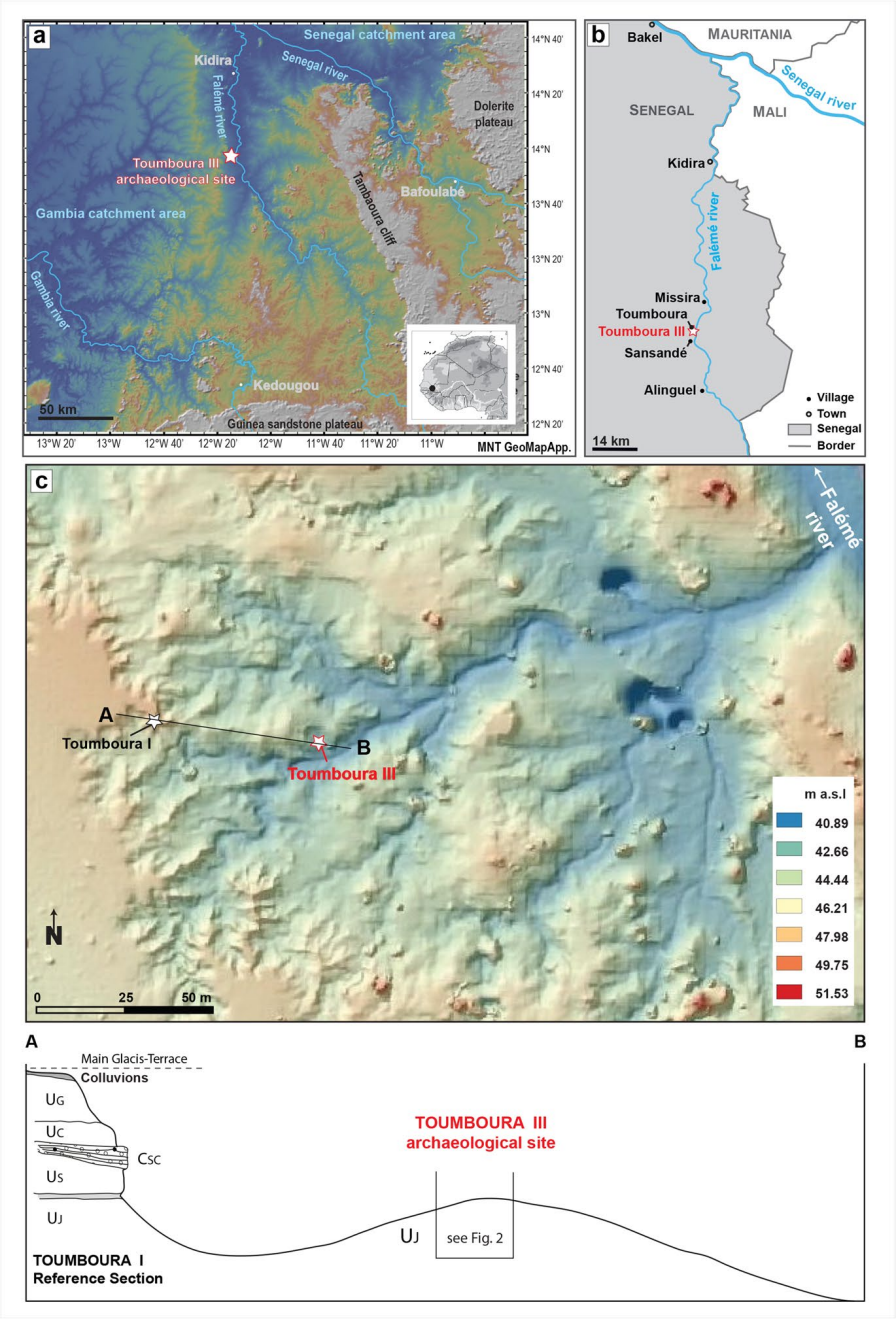
Location of the archaeological site of Toumboura III. A DTM GeoMappApp figuring the catchment areas of the Falémé River (modified after Rasse, et al., 2020). B Location of the site and the closest villages in the Falémé area (modified after Douze, et al., 2021). C Location and stratigraphic insertion of Toumboura III; top: DTM of the Toumboura area (by C. Ollier 2020); bottom: stratigraphic insertion of Toumboura III in the geomorphological reference section of Toumboura I (see Rasse et al., 2020)

TMB III, with coordinates N 13° 57′ 17.7ʺ, W 12° 12′ 46.0ʺ, is located in eastern Senegal, in the Tambacounda Region, near the Malian border (Fig. 1). The open-air site is situated on the left bank of the Falémé River, a tributary of the Senegal River, in the West Sudanian Savanna. TMB III was discovered in 2014 during the “Human Population and Paleoenvironment in Africa” international research program, which has conducted surveys and excavations in the Falémé Valley since 2011. TMB III belongs to a series of archaeological and geological sites (Toumboura I to III and North), located in a zone of intense gullying, south of the close-by village of Toumboura (Chevrier et al., 2016; Mayor et al., 2018; Rasse et al., 2020).

**Fig. 2:**
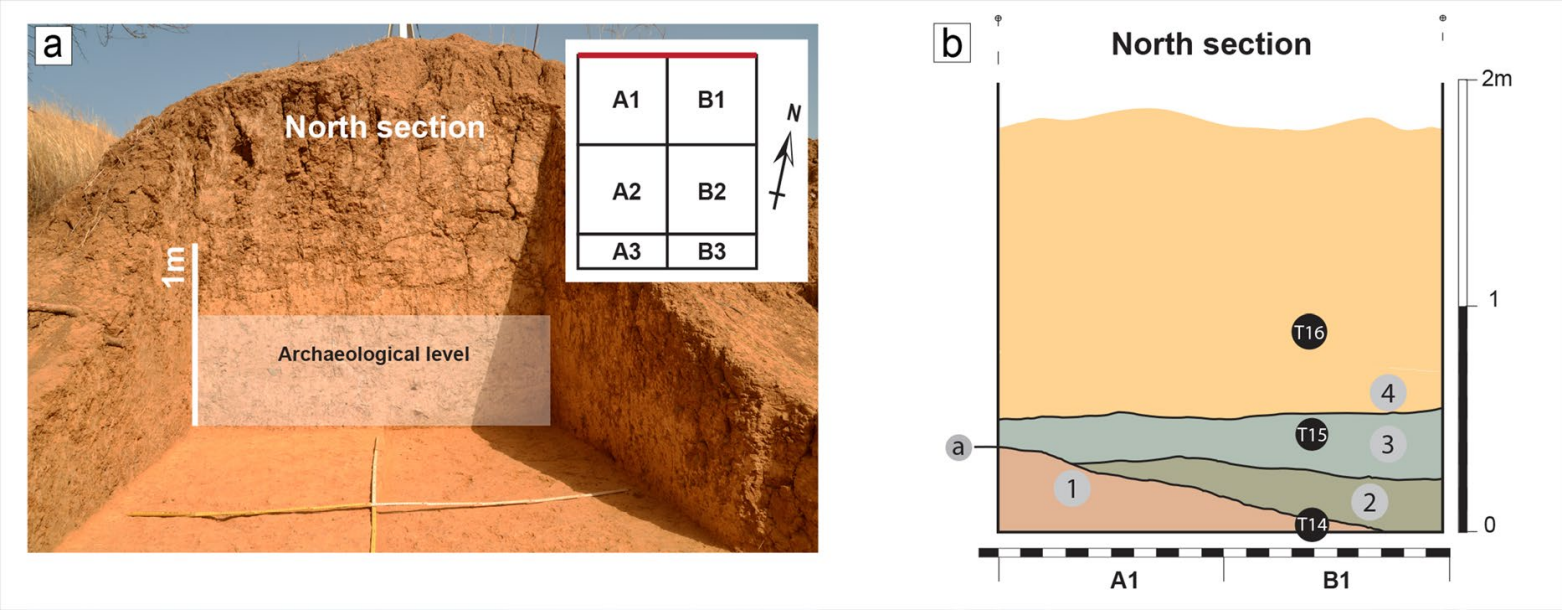
Photograph and basic stratigraphy of Toumboura III. A. View of the north section and excavation grid of Toumboura III. B. Stratigraphy of the north section of Toumboura III featuring the OSL samples (T14–16) and layers (1–4) identified within the sedimentary unit U_J_ (see chapter 3.3 for a detailed description) (photo and drawing by M. Lorenzo Martinez)

Three field campaigns were carried out at the site in 2014, 2015, and 2017. In 2014, the accumulation of archaeological remains on the surface seems to have been located at the summit of a residual mound in the eroded Pleistocene formations. Here, we launched the first excavation in the form of a test pit. The top of the archaeological horizon belongs to the general stratigraphic sequence of the Toumboura area (Rasse et al., 2020). The 2015 campaign at TMB III showed the lateral extension of the archaeological horizon and allowed the enlargement of the lithic assemblage and sampling for OSL dating. In 2017, a grid was installed in alignment with the previous campaigns (Fig. 2), and the excavation area was extended, reaching a total of 4.6 m^2^. The lithic artifacts recovered in 2017 are prioritized in this study.

### Stratigraphy

The Pleistocene formations in the fluvial context of the Falémé River Valley are governed by erosion and accumulation processes, typical for riverbanks in the Sudanian zone. They mainly correspond to overbank deposits in a seasonally flooded alluvial plain. The fine sediments were deposited by the floods of the Falémé, but the sedimentation was also fed by aeolian dust in an environment drier than the present one (Rasse et al., 2020). The TMB III stratigraphic sequence belongs to the U_J_ sedimentary unit defined in the Toumboura Formation (Fig. 1C), dated to ca. 42–23 ka (Lebrun et al., 2016, 2017). U_J_ is a homogeneous yellow silty unit, 6–5m thick, and locally constitutes the base of the Pleistocene formations (Rasse et al., 2020). At TMB III, all squares were excavated to a depth of − 230 cm below the reference zero point at the top of the mound. It is possible to distinguish the following layers, from bottom to top, within the U_J_ unit (Fig. 2):

1. Fine orange silty and sandy sediments, with many manganese inclusions and small, rare gravels. The contact with the upper units is erosive.
2. Fine silty sediments, yellow to beige, with fewer manganese inclusions.
3. Fine silty sediments, grey to beige yellow, with few millimetric manganese inclusions.
4. Silty, very fine, yellow sediments with millimetric manganese inclusions. The layer shows important bioturbation towards the top, which corresponds to the top of the mound.

The archaeological material studied here occurs in layers 1 to 4.

### Chronology

Three OSL samples (T14-T16) were taken below, within, and above the archaeological horizon (Huysecom et al., 2016; Lebrun, 2018; Lebrun et al., 2016, 2017). The OSL dating methodology and detailed data for the sample from TMB III have been published in Lebrun et al. (2016) (see also Lebrun, 2018; Lebrun et al., 2017). However, the determination of the equivalent doses has been revised, including a re-evaluation of the source calibration (Tribolo et al., 2019) and the use of the average dose model instead of the central age model (Galbraith et al., 1999; Guérin et al., 2017). Therefore, the final age estimates are slightly higher – though consistent at two sigma – than those presented in Lebrun (2018) and Lebrun et al. (2016). OSL sample T14, obtained from the base of the section in layer 1, at a depth of − 220 cm, yields an age of 40 ± 3 ka. Sample T15, from layer 3, within the archaeological horizon at ca. − 190 cm, dates to 37 ± 3 ka. Finally, sample T16 from layer 4, at a depth of − 160 cm, corresponding to the top of the archaeological horizon, provides an age of 30 ± 3 ka. Thus, the occupational phase of the site is chronologically well-framed between 40 ± 3 and 30 ± 3 ka, with layer 3 dated to 37 ± 3 ka.

## Materials and Methods

### Archaeological Sample

The excavation at TMB III has exposed a total surface of 4.6 m^2^, corresponding to squares A1, B1, A2, B2, and partially A3 and B3. The archaeological remains include lithics and worked ochre pieces, but no organic remains are preserved. This analysis focuses on the entire lithic assemblage from squares A1 and B1 excavated in 2017 by one of us (MLM). These two squares were selected as an archaeological sample because the excavation method used in this field campaign complies with modern standards, following a strict protocol. The *décapages* (arbitrary spits) of 2 cm, numbered from the top, and dry sieving of the sediments using a screen with 1-mm mesh grant a high stratigraphic resolution. The two squares towards the center of the mound yielded the highest number of artifacts and were less exposed to recent erosion.

**Fig. 3:**
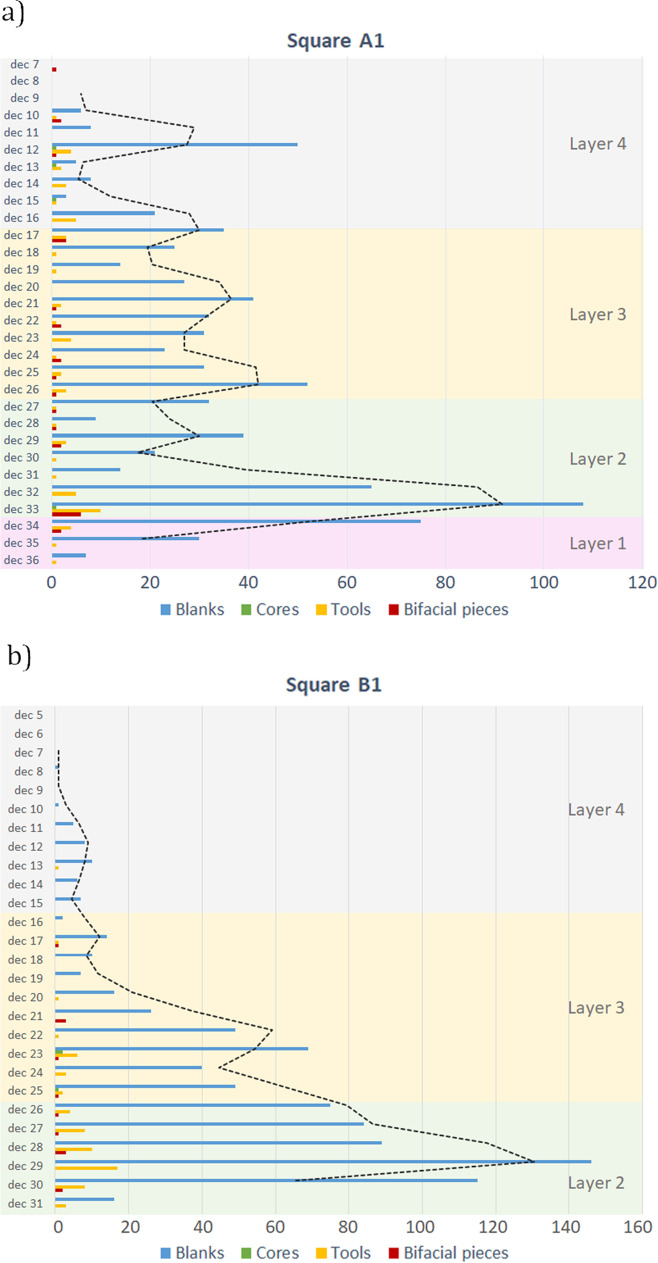
Frequency distribution of technological categories at Toumboura III: (a) Frequency distribution of technological categories by *décapage* (ca. 2 cm) in square A1; (b) frequency distribution of technological categories by *décapage* in square B1

We analyzed a total of 1830 lithics (Table 1) comprising all lithic artifacts ≥ 20 mm, and all cores, core fragments, tools, and tool fragments regardless of their size. The debitage, i.e., all pieces < 20 mm, totaled 11,226 artifacts. The fraction of debitage was quantified per square and *décapage* and according to raw material to draw further conclusions on techno-economic patterns. We consider the lithic assemblages of all layers as belonging to one technological entity due to the coherence of raw material selection, blank production, tool corpus, and the manufacture of bifacial pieces (Fig. 3a and b).

**Table 1:** General technological classification at Toumboura III

	*n*	*%*
Blanks	1,618	88.4%
Tools	165	9.0%
Cores	7	0.4%
Angular debris	35	1.9%
Heat spall	3	0.2%
Natural slab	2	0.1%
*Sub-total*	***1,830***	***100.0%***
Pieces ≥ 20 mm	1,830	14.0%
Small debitage (pieces ≤ 20 mm)	11,226	86.0%
Total (*n*)	**13,056**	**100.0%**

### Methods of Study

#### General Framework

The typological classification of blanks and cores, based on reduction strategies, complied with the commonly used terminology for the MSA (Porraz et al., 2013; Robert et al., 2003; Scerri, 2017; Tryon & Faith, 2013; Volman, 1981; Wurz, 2000). Here, we use the technological approach – *chaîne opératoire* – to analyze the lithic assemblage. This approach attempts to reconstruct the temporal sequence of the different technical stages of the knapping process, from the procurement of raw materials, through the production of blanks, tool manufacture, use, possible resharpening, and recycling to discard (Boëda et al., 1990; Leroi-Gourhan, 1964; Soressi & Geneste, 2011; Tixier, 2012). This approach is based on in-depth observations of the lithic material, and it strives to expose the intentions and technical knowledge of the craftspeople (Porraz et al., 2016; Tixier, 2012).

The implementation of the method began with grouping the artifacts by raw materials. Then, the lithics of each raw material were classified by their morphological and typo-technological characteristics to determine their positions and affinities within the operational sequence (Boëda et al., 1990; Geneste, 1991; Inizan et al., 1999; Soressi & Geneste, 2011; Tixier, 1980). Attributes and attribute combinations observed during the classification, concerning either the whole assemblage or a specific technological category, were recorded in a database and quantified to enable the application of descriptive and comparative statistical tests (Soressi & Geneste, 2011). We created an input mask of the attribute list with the software E4 (http://www.oldstoneage.com/software/e4.shtml) to enter all the data into a Microsoft Access database (see Supplementary Information, SI 1).

We used a caliper to measure the length, width, and thickness of the artifacts; a digital scale to measure weight; and a goniometer to determine exterior platform angle (EPA) and interior platform angle (IPA) of the complete and proximal preserved blanks, and the angles on the cores (see Auffermann et al., 1990; Dibble & Whittaker, 1981; Hahn, 1982; Nigst, 2012). We classified blanks with a width of ≥ 10 mm, a size ratio of length to width ≥ 2, and featuring regular, parallel edges as blades, following the definition of de Sonneville-Bordes (1960), Crabtree (1972), Hahn (1977), and Inizan et al. (1999). Moreover, we distinguished elongated flakes, which exhibit parallel dorsal ridges and regular morphologies, but have a length to width ratio between 1.5 and > 2 (de Sonneville-Bordes, 1960; Moreau, 2009). We followed the diagnostic stigmata list of Pelegrin (2000) and Soriano et al. (2007) concerning the identification of the knapping techniques. However, we concede the need for controlled experiments to ideally establish unambiguous criteria for sandstone with regard to different techniques. Ultimately, to strengthen our proposed hypotheses, we conducted univariate descriptive and comparative tests using the statistics software Past (Hammer et al., 2001).

#### Raw Material Identification

Raw material selection is the first implicit identifiable step in the *chaîne opératoire* approach. In our study, raw material characterization is based on previous works in the Falémé Valley (Chevrier et al., 2020; Douze et al., 2021; Rasse et al., 2020) and ongoing petrographic analyses conducted at the University of Geneva. The assessment of their availability and provenance in the landscape relies on field observations in Toumboura, more broadly along the Falémé River Valley, and on the geological map of eastern Senegal (Goujou et al., 2010). Depending on the rock type, different types of raw material sources were identified, including primary outcrops and riverbank deposits. Natural surfaces observed on the lithic artifacts were subdivided into different categories to evaluate their origin. The combined description of the petrography and the natural surfaces guided the assessment of the raw material selection patterns.

#### Adapted Method for Bifacial Pieces

We noted the predominance of bifacial implements and shaping by-products from the observations made during the excavation and the preliminary techno-typological analysis by Chevrier and Leplongeon (Chevrier et al., 2016; Huysecom et al., 2016) and adapted our study protocol accordingly. Following Inizan et al. (1999), we define shaping (*façonnage*) as a sequence of knapping operations aiming to fabricate one single artifact by fashioning the raw material in compliance with the desired size and shape, such as a handaxe, a Still Bay point, or a bifacially shaped arrowhead. Bifacial pieces, also referred to as bifacial tools or bifacials, include all tools that exhibit continuous shaping on both dorsal and ventral surfaces along the same portion of an edge (Inizan et al., 1999).

We created a form for all complete or broken bifacial pieces (see SI 2) to record details of the raw material and morphometric characteristics. The types of macro-fractures were recorded in compliance with Fischer et al. (1984). Moreover, following Villa et al. (2009), each bifacial tool was classified according to its manufacture phase by using attributes such as the portion of the unmodified surface, regular outline of tip and base, bifacial symmetry, and bilateral symmetry. Villa et al. (2009) divided the manufacturing sequence into four phases: (1) initial shaping; (2) advanced shaping; (3) finished product; and (4) recycling, with a subdivision of phase 2 into 2a and 2b. Phase 2a shows removals resulting from hard and soft hammer percussion, some residual cortex, and mostly irregular outlines and profiles. In contrast, phase 2b has exclusively soft hammer scars, fewer cases of residual cortex, and mostly regular outlines and profiles.

Furthermore, we described chronology and organization of the shaping with consideration to orientation, size, morphology, delineation, angle, presence of counter-bulbs, and the profile of the flakes to reconstruct the sequence(s) of manufacture, including the hierarchization and technique(s), and the variability of the finalized bifacial tools. We systematically created diacritic diagrams, which represent schematic drawings to underline the still visible series of knapping actions preserved on the artifact (Dauvois, 1976; Inizan et al., 1999; Soressi, 2002).

We used a techno-functional approach to provide a theoretical functional characterization of the tool by identifying the three parts of the artifact working in synergy, defined by an ensemble of technical elements that co-exist (see Boëda, 1997, 2001, 2013; Bourguignon, 1997; Chevrier, 2012; Lepot, 1993; Soriano, 2000, 2001). These three parts relate to : (1) the active part, which is meant to be in contact with the worked material; (2) the passive part, which is intended to facilitate the handling of the tool; and (3) the intermediate part passing on the transmitted energy from the active to the passive part during an action. Additionally, we documented the location and distribution of macro-removals and micro-removals on the different parts of the tools at low magnification (× 10– × 40) to characterize the supposedly intentional (i.e., technological) and unintentional (i.e., functional) traces. Moreover, we used an Olympus SZX10 stereo microscope for a high-resolution visualization and documentation of specific features on bifacial pieces. Finally, we created 3D models of three diagnostic pieces (see SI 3).

#### Adapted Method for Shaping Flakes

We studied shaping flakes, which exhibit technical and morphological characteristics reflecting key changes in the bifacial tool production process, from roughout to finished product. Following and elaborating on Soriano et al. (2009) and (2015), we discerned three types of shaping flakes related to: (1) early/initial blank shaping; (2) advanced shaping; or (3) final shaping (Table 2). Our analysis further identified an earlier phase 2a and a later phase 2b of the advanced shaping (Table 2). In addition, we identified diagnostic elements indicating a phase of tool restructuration during the shaping process (R). The manufacturing phases 1 to 3 correspond to the stages between the bifacial pieces and the shaping flakes. In contrast, phase 4 (recycling), as defined by Villa et al. (2009), is only observed on the bifacial tools themselves. The phase of restructuration (R) seen through the shaping flakes is not chronologically sequential, but can occur at any point during manufacture, serving to correct knapping errors or to modify the structure of the tool.

**Table 2:** Attributes of the shaping flake types at Toumboura III

Early phase	2a. Advanced phase	2b. Advanced phase	3. Final phase	R. Restructuration shaping
*Fixed characteristics:* Platform type: plain or cortical EPA: high (≥ 60°) Morphology: fan-shaped (trapezoidal) or rectangular Number of dorsal removals: **low** (*n* = 0–4) Direction of dorsal scars: unidirectional Cortex, natural surface or old ventral surface on dorsal surface distal part	*Fixed characteristics:* Presence of poorly developed bulb or no bulb Morphology: fan-shaped (trapezoidal) or rectangular	*Fixed characteristics:* Platform type: plain, dihedral, or faceted Presence of lip Presence of poorly developed bulb or no bulb Morphology: fan-shaped (trapezoidal) or rectangular No cortex, natural surface or old ventral surface on dorsal surface	*Fixed characteristics:* Platform type: plain, dihedral or faceted Presence of lip No bulb Dorsal reduction: preparation of overhang with short removals Morphology: fan-shaped (trapezoidal) Number of dorsal removals: medium (*n* = 2–5) Direction of dorsal scars: unidirectional No cortex, natural surface or old ventral surface on dorsal surface	*Fixed characteristics:* Platform type: faceted Platform shape: oval or lunate Presence of lip Presence of poorly developed bulb or no bulb Porsal reduction: no preparation of overhang Morphology: fan-shaped (trapezoidal) Number of dorsal removals: low (*n* = 1–4) Direction of dorsal scars: unidirectional Dorsal scars: flat and without contre-bulb Profile: frequently rectilinear
*Variable characteristics:* Platform shape: oval, quadrangular, triangular, lunate, or winged Presence of prominent bulb Thickness: medium to thick (≥ 3– < 30 mm) Profile: rectilinear, slightly curved or curved Cortex, natural surface or old ventral surface secant, parallel or subparallel to platform	*Variable characteristics:* Platform type: frequently plain or cortical Platform shape: oval, quadrangular, triangular, or winged EPA: more frequently low Presence of lip Number of dorsal removals: varying (*n* = ≥ 1) Direction of dorsal scars: mainly bidirectional or orthogonal Rarely cortex, natural surface or old ventral surface on dorsal surface distal part Thickness: medium to thick (≥ 3– < 20 mm) Profile: frequently curved	*Variable characteristics:* Platform shape: oval, quadrangular, triangular, winged or linear EPA: frequently low (> 80°) Dorsal reduction: mainly preparation of overhang with short removals Number of dorsal removals: varying (*n* = ≥ 2) Direction of dorsal scars: mainly Bidirectional or orthogonal Thickness: thin (> 2– < 7 mm) Profile: frequently curved	*Variable characteristics:* Platform shape: oval, quadrangular, triangular, winged or linear EPA: frequently low (> 80°) Thickness: thin (> 2– ≤ 6 mm) Profile: frequently curved	*Variable characteristics:* EPA: varying (≥ 50°– ≤ 90°) Thickness: medium (> 3– < 11 mm)
Flake type: Cortical flake, Kombewa, *débordant, dos limité*	Flake type: Flake, cortical flake, Kombewa,* débordant, dos limité*	Flake type: Flake	Flake type: Flake	Flake type: Flake

As is the case with the entire bifacial shaping process, we observe fluidity between the phases of the bifacial shaping by-products rather than abrupt transitions. A single attribute is not sufficient to qualify a classification; only the combination of attributes is decisive (Soriano et al., 2009, 2015). Every type stands out by both fixed and variable characteristics. Some of the products were classified as atypical shaping flakes (Table 3). Though fixed (typical) characteristics are present, these shaping flakes either lie at the extreme variation range or are missing their proximal parts. 

Finally, we acquired data on the proportion of shaping flakes among the fractions of small debitage to evaluate the intensity of onsite tool manufacturing and recycling. For this purpose, we selected *décapages* 26 and 33 of A1, which demonstrate the highest frequencies of small debitage in this square. For the pieces ≥ 20 mm, we identified the shaping flakes according to the attributes established by Soriano et al. (2009, 2015).

**Table 3:** Blank categories at Toumboura III

	*n*	*%*
Blades	6	0.4%
Elongated flakes	8	0.5%
Indifferent flakes	480	29.7%
Bipolar flakes	3	0.2%
Preferential flake	1	0.1%
Core tablet	1	0.1%
Crested flakes	5	0.3%
*Débordants* (core edge products)	27	1.7%
*Dos limité*s (pseudo-Levallois points)	4	0.2%
Kombewa flakes	12	0.7%
Notch resharpening flake	1	0.1%
*Shaping flakes*	***724***	***44.7%***
*Atypical shaping flake*	***346***	***21.4%***
Total	**1,618**	**100.0%**

## Results

### Taphonomy

Information gathered from the lithics allows the assessment of the taphonomic background of the TMB III assemblage. The archaeological finds are vertically distributed over 40 cm (layers 1–4). The majority of the artifacts from squares A1 and B1 come from layers 2 and 3, which seem to coincide with the original archaeological horizon before post-depositional vertical processes and colluvial inputs on the margin of the floodplain occurred.

Square A1 yielded 904 lithic artifacts (see SI 4a). Layers 2 and 3 exhibit the highest proportions with 37.6% (*n* = 341) and 35.4% (*n* = 321), respectively. The small debitage, i.e., all pieces < 20 mm, forms a total of 6090 artifacts, of which the majority also comes from layer 3 (49.1%) and layer 2 (27.3%). Square B1 yielded 926 lithics, from layers 2 to 4 (see SI 4b). Layer 3 has a total of 305 pieces (32.9% of the total). Layer 2 demonstrates the highest proportion at 62.9% (*n* = 582). The small debitage encompasses 5136 artifacts, peaking in layers 3 (50%) and 2 (42.1%).

It was revealed during the excavation that the finds often show oblique or even vertical inclinations with different orientations. In addition, some of the artifacts have concretions on their surfaces, and 56.1% of the lithics show edge damage in the form of random scarring. In terms of depositional processes, the archaeological assemblage is stratified, horizontally transported only over short distances, possibly by water, and rapidly covered by fine-grained sediments. It is also minimally reworked by post-depositional processes, possibly due to vertisolization and bioturbation, the latter probably having caused the vertical dispersion. However, small debitage is strongly represented, the state of the artifact preservation is excellent (i.e., 70% of the pieces with relatively fresh edges), and uniformity of raw material and technological composition is apparent. The minor artifact scattering on the surface and the low material density towards the east indicate a spatially limited occupation (Chevrier et al., 2016; Huysecom et al., 2016; Mayor et al., 2018).

### Lithic Technology

This section presents the findings on the raw material selection, reduction strategies, knapping techniques, blank output, tool production, shaping, and resharpening. The lithic assemblage consists of a high frequency of blanks, a large proportion of retouched elements (9% of the assemblage), and an extremely low number of cores (Table 1). The blank composition comprises a prevalence of shaping by-products (Table 3). Apart from the frequent occurrence of side scrapers and notched pieces, the tool corpus demonstrates a strong emphasis on a variety of bifacial pieces (Table 4).

**Table 4:** Typological corpus at Toumboura III (*presentation of different phases of manufacture in Table 8)

	*n*	*%*
Side scrapers	68	41.2%
Notched pieces	25	15.2%
Denticulates	10	6.1%
*Pièces esquillées* (splintered pieces)	8	4.8%
End scrapers	4	2.4%
Composite tools (notched piece and side scraper)	4	2.4%
Multi-side scrapers	3	1.8%
Becs	2	1.2%
Multi-notched pieces	1	0.6%
Unifacial point	1	0.6%
Bifacial pieces*	***39***	***23.6%***
Total	**165**	**100.0%**

#### Raw Material Economy

The toolmakers of TMB III mostly exploited locally available raw materials, especially less than 5 km from the site (Douze et al., 2021; Rasse et al., 2020). We discerned seven rock categories within the assemblage: sandstone, silexite, quartzite, quartz, granite, limestone, and rhyolite. The knappers primarily used blue-grey sandstone of different qualities (Table 5; also Douze et al., 2021). To a lesser extent, silexite (also called chert) of varying colors (but mainly blue-green) was used, followed by quartzite and quartz. Three single blanks are made on limestone, granite, and rhyolite, whereby the limestone piece is a shaping flake. Three cortex types were distinguished: alluvial, weathered, and natural fissure surface. The cortical pieces made on sandstone, silexite, and quartzite exhibit all three cortex types, though sandstone involves mostly artifacts with weathered cortex or natural surfaces originating from (sub)primary contexts. Quartz lithics, with alluvial cobble cortex, were probably gathered from the riverside.

**Table 5:** Frequency of raw materials per technological classification at Toumboura

Technological classification	Sandstone		Silexite		Quartzite		Quartz		Others	
	*n*	%	*n*	%	*n*	%	*n*	%	*n*	%
Blanks	1,551	95.9%	43	2.7%	13	0.8%	8	0.5%	3	0.2%
Tools	126	76.4%	33	20.0%	4	2.4%	2	1.2%	-	0.0%
Cores	-	0.0%	5	71.4%	-	0.0%	2	28.6%	-	0.0%
Angular debris	33	94.3%	2	5.7%	-	0.0%	-	0.0%	-	0.0%
Heat spall	3	100.0%	-	0.0%	-	0.0%	-	0.0%	-	0.0%
Natural slab	1	50.0%	1	50.0%	-	0.0%	-	0.0%	-	0.0%
*Sub-total*	***1,714***	***93.7%***	***84***	***4.6%***	***17***	***0.9%***	***12***	***0.7%***	***3***	***0.2%***
Small Debitage	10,276	91.5%	796	7.1%	73	0.7%	81	0.7%	-	0.0%
Total	**11,990**	**91.8%**	**880**	**6.7%**	**90**	**0.7%**	**93**	**0.7%**	**3**	**0.0%**

The widths of the sandstone blanks (*n* = 664; median = 23.9 mm; mean 26 mm) are significantly larger than those of the silexite blanks (*n* = 21; median = 19.5 mm; mean = 19.8 mm) (Mann–Whitney *U* test, *U* = 3548, *p* = 0.0001258). This difference in size could indicate that (1) the silexite is only available in smaller volumes; (2) only smaller silexite volumes were selected even though they existed in larger volumes; or (3) the smaller silexite products are the result of the many internal cracks and fissure planes, which, as a result, only make small volumes of homogeneous raw material suitable for knapping. The third hypothesis seems most likely, as we often observed natural fissure surfaces on silexite products.

Preferential raw material selection, according to the different production goals, is also stressed by the absence of cores made on sandstone and quartzite, while they occur in silexite (6%) and are common in quartz (16.7%). Conversely, high frequencies of shaping flakes occur among sandstone (67.2%) and quartzite (69.2%) blanks (Table 6). They are also common among silexite blanks (39.5%) but significantly less frequent within the quartz blank population (12.5%). Thus, sandstone and quartzite are mainly processed by shaping, silexite by both shaping and core reduction, and quartz predominantly by core reduction. These explain why the tool-to-blank ratio (see Dibble & McPherron, 2006) for sandstone shows a low overall degree of modification (0.01) compared to silexite (0.2), quartzite (0.5), and quartz (0.2).

**Table 6:** Frequencies of raw material types in shaping flakes, in bifacial pieces and in shaping flakes ≤ 20 mm from *décapages* 26 and 33 at Toumboura III

Technological categories	Sandstone		Silexite		Quartzite		Quartz		Others	
	*n*	%	*n*	%	*n*	%	*n*	%	*n*	%
Shaping flakes	1042	97.4%	17	1.6%	9	0.8%	1	0.1%	1	0.1%
Bifacial pieces	16	41.0%	19	48.7%	3	7.7%	1	2.6%	-	0.0%
Shaping flakes < 20 mm (square A1, décapage 26)	79	86.8%	11	12.1%	1	1.1%	-	0.0%	-	0.0%
Shaping flakes < 20 mm (square A1, décapage 33)	47	83.9%	7	12.5%	2	3.6%	-	0.0%	-	0.0%

Sandstone seems to be subjected to two behavioral factors regarding the raw material economy: (1) long and intense shaping events and (2) the absence of the first phases (initiation and early preparation) in the operational sequence. Regarding silexite and quartzite, both the import of prepared artifacts to the site and the export of finished end products possibly played a role. Finally, silexite demonstrates the highest ratio of small debitage to single lithic finds at 9:1, highlighting onsite knapping activities, probably often related to shaping and retouching, and the small size of the original volumes. On the contrary, quartzite exhibits the lowest ratio at 8.1:1.9, indicating less production, manufacturing, and resharpening at the site. Thus, raw material economy, with regard to the lithic production goals, seems to be a dynamic process at TMB III.

#### Core Reduction Method

**Fig. 4:**
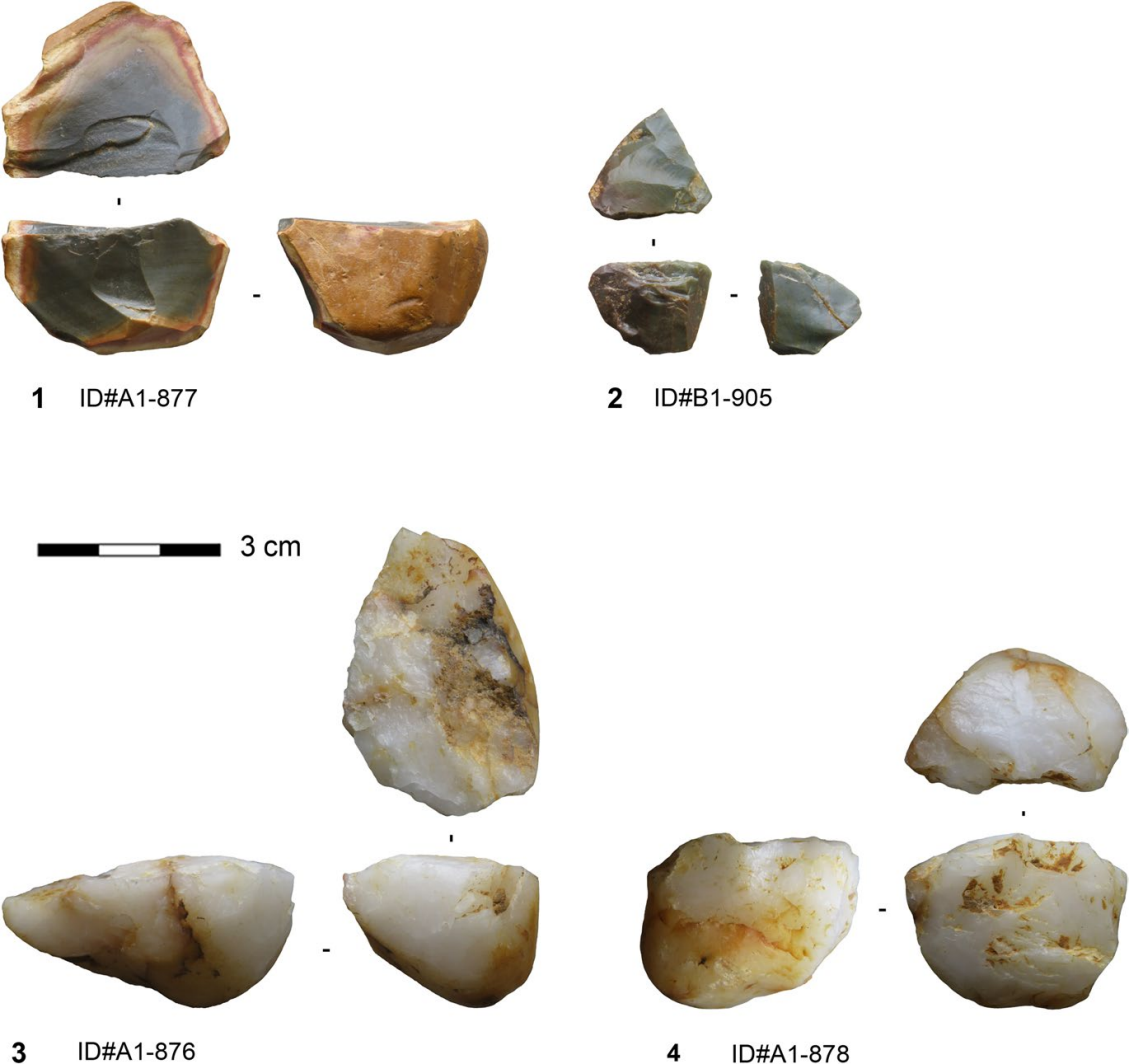
Algorithmic cores from Toumboura III. 1–2 Silexite cores; 3–4 Quartz cores (photos by V. C. Schmid)

Core reduction plays a minor role in the assemblage (Table 1). The seven cores are exclusively made on silexite and quartz. They are of small size, with a mean length of 27 mm (median = 25.6 mm), mean width of 25 mm (median = 30.8 mm), and a mean thickness of 17.3 mm. Nodules, slabs (for silexite), and flakes were selected as original morphologies. The geometry of the cores is consistent with a volumetric conception and concurs with a unidirectional single platform (Figs. 4.1,3-4) and prismatic morphologies (Fig. 4.2). Removal surfaces are quadrangular. The core back is either covered by cortex or formed by the ventral face of the original blank. The core bases and at least one lateral side show cortex coverage in all cases. Striking platforms are plain, cortical, or formed by the scar of a previous removal. Three cores exhibit a single striking platform and one corresponding removal surface, while one core has two independent striking platforms and removal surfaces. Two cores exhibit a previous removal surface converted into a striking platform for the last reduction sequence. The core with bidirectional scars on one removal surface has two opposed striking platforms, and the removals from each striking platform indicate an independent sequence and a clear chronological separation. The reduction strategy is rather simple and could be described as algorithmic (Boëda, 1997; Forestier, 1993; Soriano & Huysecom, 2012). This entails an unchangeable sequence of parallel unidirectional removals, repeated as often as possible depending on the available convexities, to obtain a series of small blanks. The rather unstandardized reduction sequence does not require high preparation effort, as the adopted volume provides naturally, to a certain extent, the technical prerequisites to control the exploitation (Boëda, 2013). The last operational phases identified on the cores reveal the production of small flakes and elongated flakes. The dimensions and the severity of knapping accidents, primarily hinges, seem to have led to their discard.

#### General Presentation of the Bifacial Technology

One outstanding feature of the TMB III assemblage is the bifacial technology, as evident in the bifacial tools and the high number of shaping flakes (66.1%). The total of 39 bifacial pieces is mainly manufactured on silexite (*n* = 19) and sandstone (*n* = 16), followed by quartzite (*n* = 3) and quartz (*n* = 1). Most of the bifacials (*n* = 33) are fragmented. The complete pieces have a mean length of 57.9 mm (median = 50.9 mm), mean width of 24.7 mm (median = 22.6 mm), and mean thickness of 10.1 mm (median = 8.7 mm) (Table 7). Considering the ratio of shaping flakes to bifacial pieces, sandstone shows a much higher value (65.1) than quartzite (3), quartz (1), and silexite (0.9) (Table 6). As previously stated, this noticeable divergence may reflect differential techno-economic organizational patterns related to import and export of finished or semi-finished products, bifacial tool-type variability, and/or intensities of shaping and resharpening according to the raw materials used. The same pattern concerning the raw material representation emerges when considering the shaping by-products among the small debitage products from *décapages* 26 and 33 (Table 6).

**Table 7:** Descriptive statistics of length, width, thickness, and weight of bifacial pieces at Toumboura III

	Length (mm)	Width (mm)	Thickness (mm)	Weight (g)
n	7	31	39	5
Min	30.0	8.7	2.3	6.0
Max	101.3	63.5	27.2	204.6
Sum	405.3	765.0	393.0	353.6
Mean	57.9	24.7	10.1	70.7
Std. error	9.6	2.4	1.0	37.1
SD	25.5	13.3	6.0	83.0
Median	50.9	22.6	8.7	31.1

#### The Three Types of Bifacial Pieces

The bifacial pieces, comprising 23.6% of the formal tools (Table 4), encompass all phases of the manufacturing sequence from initial to advanced shaping, completing, and resharpening the final tool (Table 8). However, we observed differences concerning the raw materials. The pieces made on silexite demonstrate all phases and subphases of the manufacturing process. Initial (phase 1) and early advanced (phase 2a) shaping elements, as well as finished (phase 3) tools, are present among the sandstone bifacial tools, while late advanced pieces (phase 2b) are missing. All three quartzite bifacials are finished products, and the only quartz bifacial tool corresponds to the phase of advanced shaping. Moreover, we differentiated three objectives of the bifacial production: (1) bifacial pieces with planoconvex cross sections; (2) bifacial points; and (3) bifacial pieces with a natural back. These different types vary in the used rock types, technological and possibly functional properties, and morphometrics.

**Table 8:** Frequency of different phases of manufacture of bifacial pieces per raw material at Toumboura III

Raw materials	Phase 1		Phase 2		Phase 2a		Phase 2b		Phase 3		Total	
	*n*	*%*	*n*	*%*	*n*	*%*	*n*	*%*	*n*	*%*	*n*	*%*
Sandstone	5	31,3%	-	0,0%	2	12,5%	-	0,0%	9	56,3%	16	100%
Silexite	2	11,8%	-	0,0%	2	11,8%	3	17,6%	10	58,8%	17	100%
Quartzite	-	0,0%	2	40,0%	-	0,0%	-	0,0%	3	60,0%	5	100%
Quartz	-	0,0%	1	100,0%	-	0,0%	-	0,0%	-	0,0%	1	100%
Total	**7**	**17,9%**	**3**	**7,7%**	**4**	**10,3%**	**3**	**7,7%**	**22**	**56,4%**	**39**	**100%**

#### Bifacial Pieces with Planoconvex Cross Sections

**Fig. 5:**
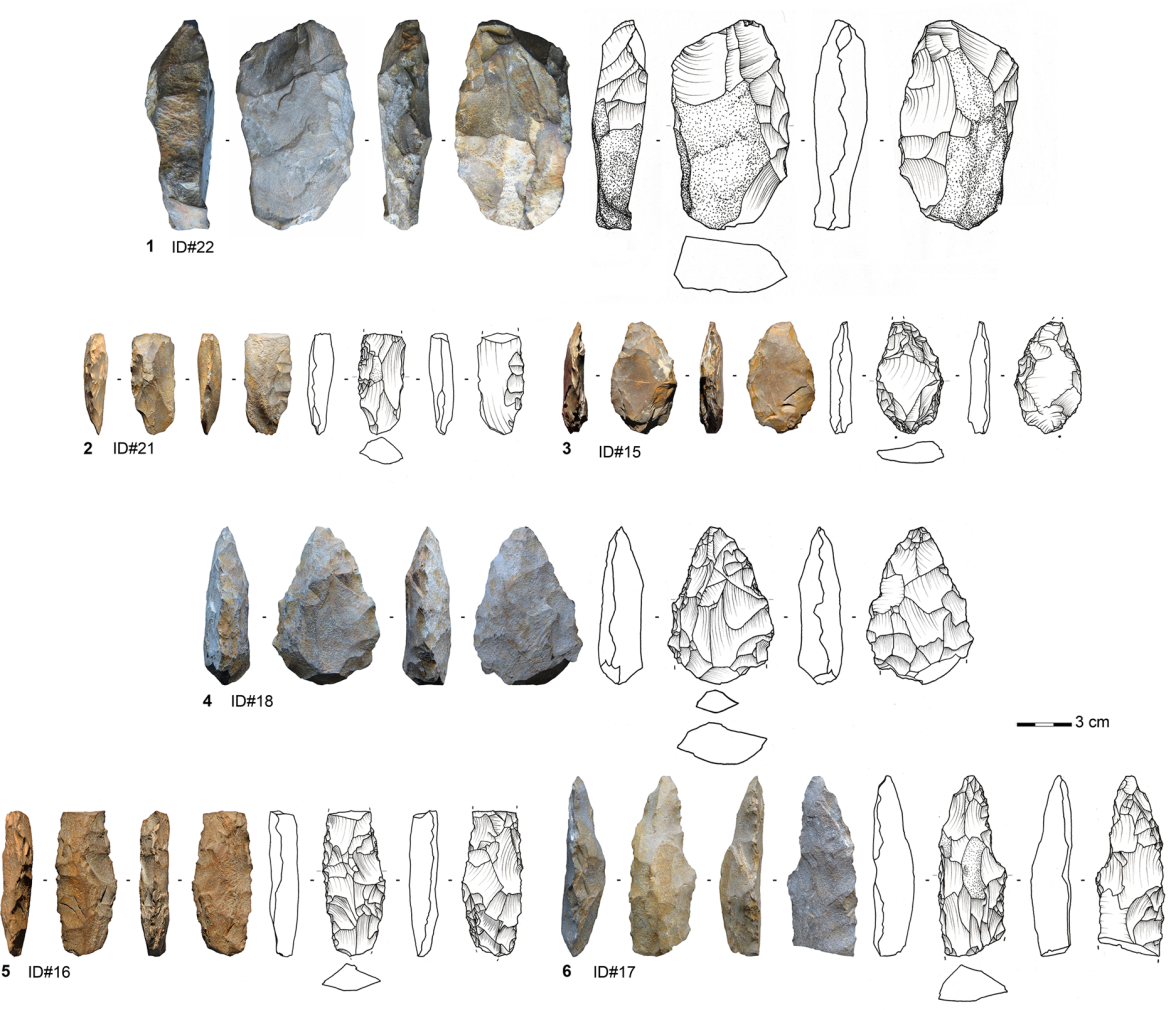
Bifacial pieces with planoconvex cross sections made on sandstone from different manufacturing phases at Toumboura III. 1–2 from manufacturing phase 1; 3 from manufacturing phase 2a; 4–6 from manufacturing phase 3 (photos by V. C. Schmid; drawings by H. Würschem)

**Fig. 6:**
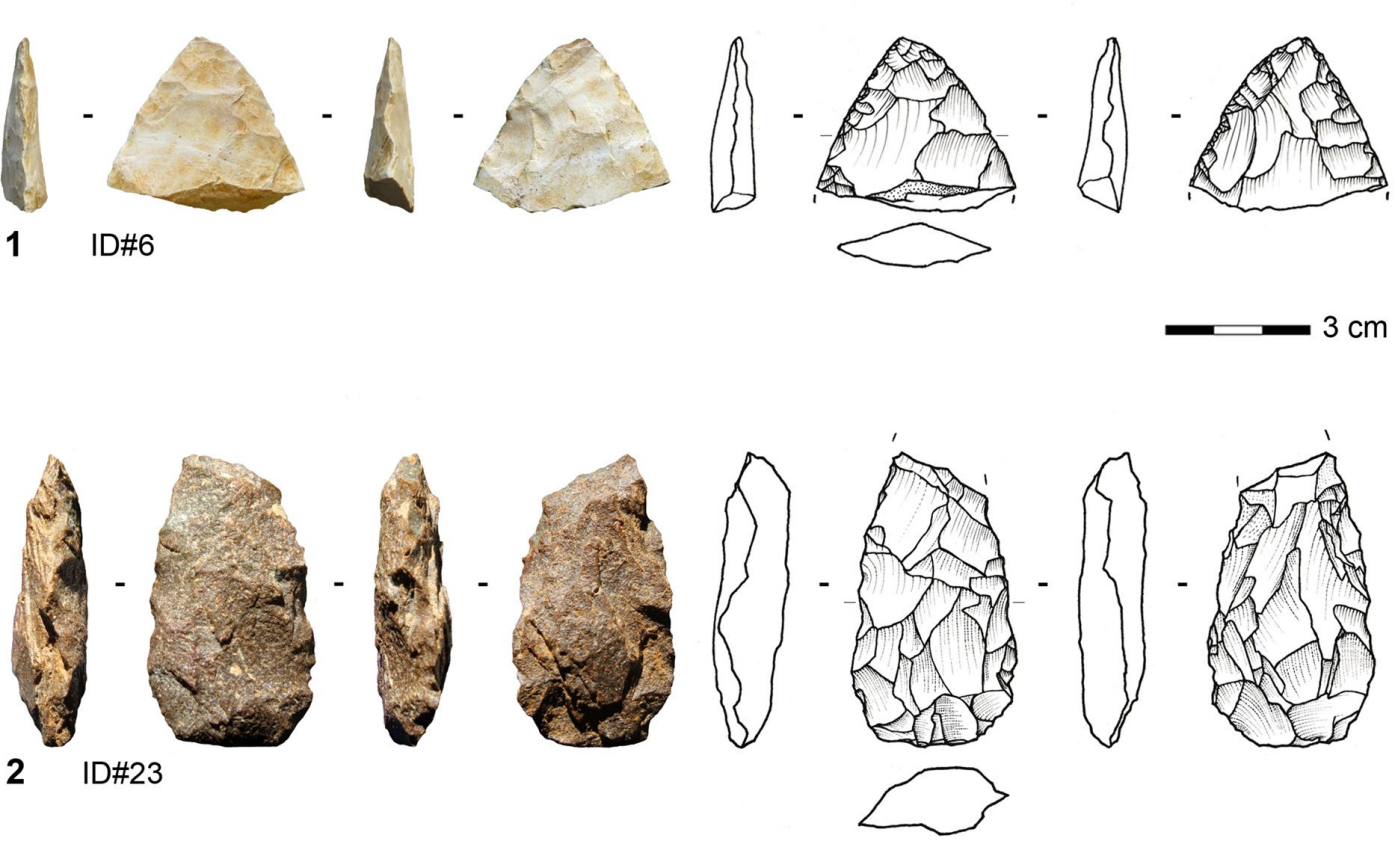
Bifacial pieces with planoconvex cross sections made on quartzite from Toumboura III. All from manufacturing phase 3 (photos by V. C. Schmid; drawings by H. Würschem)

Most of the bifacial pieces made on sandstone and all those made on quartzite (*n* = 18 in total) have planoconvex cross sections and show similarities in the manufacturing process and morphometric and techno-functional features (Figs. 5 and 6). Three pieces are completely preserved, and most of the fragmented bifacials exhibit snap fractures, i.e., bending fractures with straight or curved profiles, often appearing during manufacture. The majority of these bifacial tools (*n* = 10) are finished products (Figs. 5.4-5 and 6), followed by elements from the initial shaping (*n* = 5) (Fig. 5.1-2) and the early advanced shaping phase (*n* = 2) (Fig. 5.3). The lack of pieces from the late advanced shaping phase (2b) suggests that knappers either discarded the preforms very early on due to failures to establish the essential configuration or managed, because of the reworking prospects, to generate a usable tool anyways.

The early shaping bifacials show that flakes and slabs were used as volumes to be transformed into bifacial tools. Due to the morphometric premises, the selection of flakes entails a directedness towards more elongated and thinner bifacials than those coming from slabs. All of these products exhibit at least 20% cortex coverage, natural surface, or unmodified ventral/dorsal surface of the original blank. The knappers used primarily hard hammer percussion in the early shaping phase. The only completely preserved bifacial piece from the initial shaping phase (Fig. 5.1) has a length of 101.3 mm and a weight of 204.6 g. In comparison, the only completely preserved finished bifacial tool exhibits a length of 80.6 mm and a weight of 97.4 g, indicating a guideline for size and mass. The width and thickness values are higher in the early shaping than in the final shaping bifacials (Table 9). The finished products made on sandstone and quartzite show a significantly greater tool size than those made on silexite and quartz, attested by maximum width (Mann–Whitney *U* test, *U* = 4, *p* = 0.0042353) and maximum thickness (Mann–Whitney *U* test, *U* = 5, *p* = 0.0019977).

**Table 9:** Descriptive statistics of width and thickness of different manufacturing phases of bifacial pieces with planoconvex cross sections at Toumboura III

	Width (mm)			Thickness (mm)		
	*Early shaping*	*Advanced shaping*	*Final shaping*	*Early shaping*	*Advanced shaping*	*Final shaping*
n	5	2	10	5	2	11
Min	24.0	22.6	11.6	11.3	8.7	3.4
Max	63.5	37.5	55.0	27.2	12.5	23.0
Sum	191.0	60.1	300.2	87.6	21.2	130.1
Mean	38.2	30.1	30.0	17.5	10.6	11.8
Std. error	6.7	7.5	4.1	2.8	1.9	2.1
SD	14.9	10.5	13.0	6.3	2.7	6.9
Median	34.6	30.1	32.7	15.9	10.6	9.8

The morphologies of the finished bifacials (phase 3) range, depending on the degree of resharpening and the shape of the original blank, from pointed oval (Fig. 5.4) to elongated narrow with parallel edges (Fig. 5.6). The majority demonstrate planoconvex cross sections or a combination of plane and convex of each surface of the bifacial piece. Toolmakers started the production on the flat surface with large removals and continued on the convex surface. The large removals of the flat surface are partially strongly overprinted and reduced by several actions of resharpening or a long shaping event on the convex surface implied by the steepness and stepped morphology of the last shaping scars. These removals indicate a sequential and hierarchical procedure of shaping that enables a great reworking or resharpening potential on the tool without altering its entire structure (Boëda, 1997, 2001; Nicoud, 2011; Soriano et al., 2015). The shallow negative bulbs and the shape of the removals imply the use of marginal soft hammer percussion for the advanced phases of the shaping process.

The finished bifacials have either pointed or slightly convex-shaped tips, forming their distal transformative end (i.e., potential working edge of the tool). The outline of these transformative parts is maintained by reoccurring resharpening events. The opposite basal ends show an elliptic or quadrangular symmetric morphology. The separation of the working edge and supposedly prehensile part is marked by changes in the profile, delineation, angle, and morphology of the removals. The extent of retouched working edges appears virtually identical on both edges of the finished bifacials, suggesting axial hafting. The edge angles of both lateral edges range between 40° and 78° with a mean of 58.7° (median = 58°) suited for both vertical (piercing, slicing, or stabbing) and horizontal (scraping, whittling, or smoothing) functioning (Soressi, 2004). We identified no direct evidence of hafting, such as polish, scarring, or bright spots (Rots, 2010), although one piece shows smoothing of the prominent ridges from the base to the mid-part, where the outline starts to be more intensely worked. This resulted most likely from repeated but limited movement in the haft (Fig. 5.6). The configuration and design of these bifacial pieces indicate they would have been used as pointed cutting tools rather than spear tips.

#### Bifacial Points

**Fig. 7:**
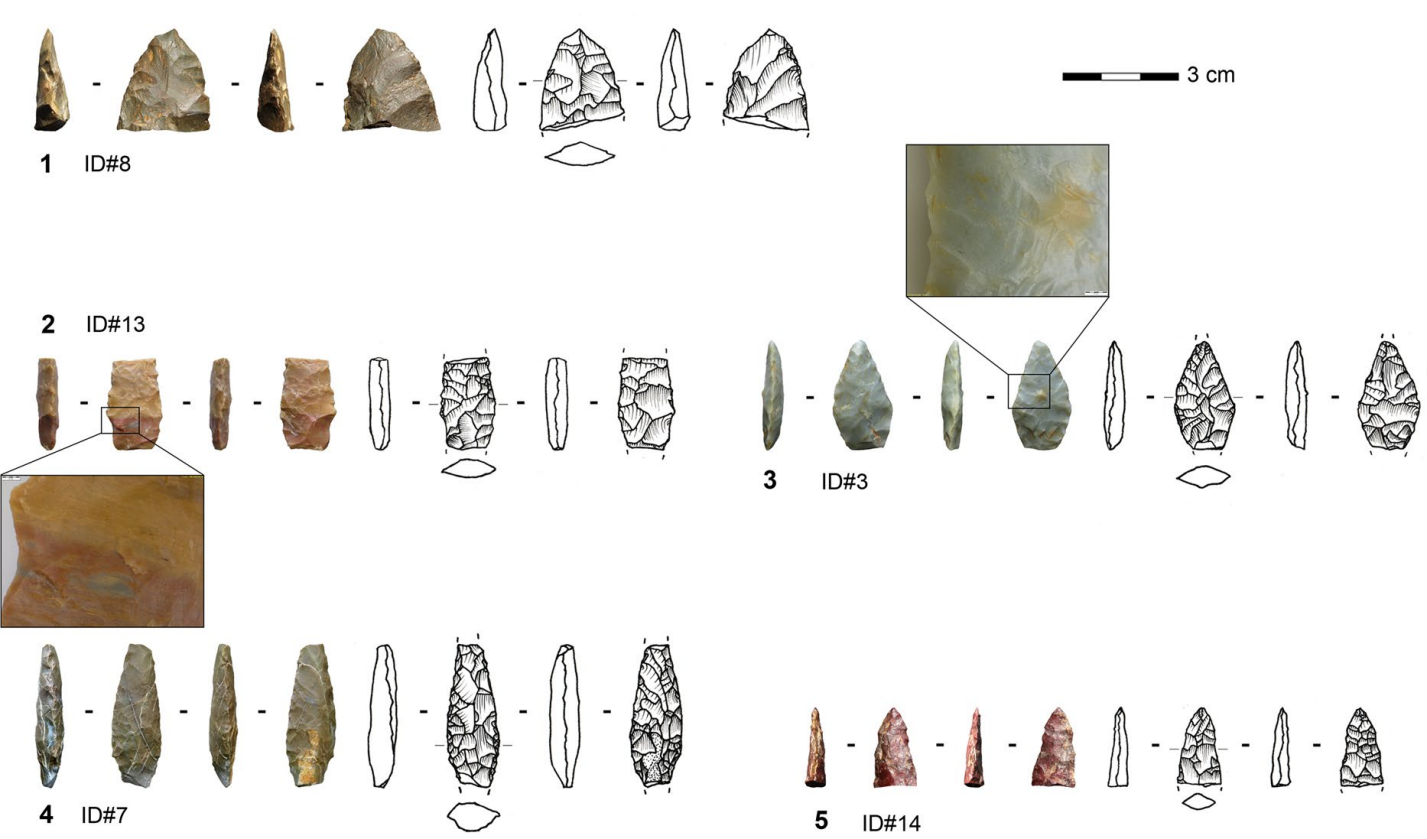
Bifacial points made on silexite from different manufacturing phases from Toumboura III. 1 from manufacturing phase 2b; 2–5 from manufacturing phase 3 (photos by V. C. Schmid; drawings by H. Würschem)

**Fig. 8:**
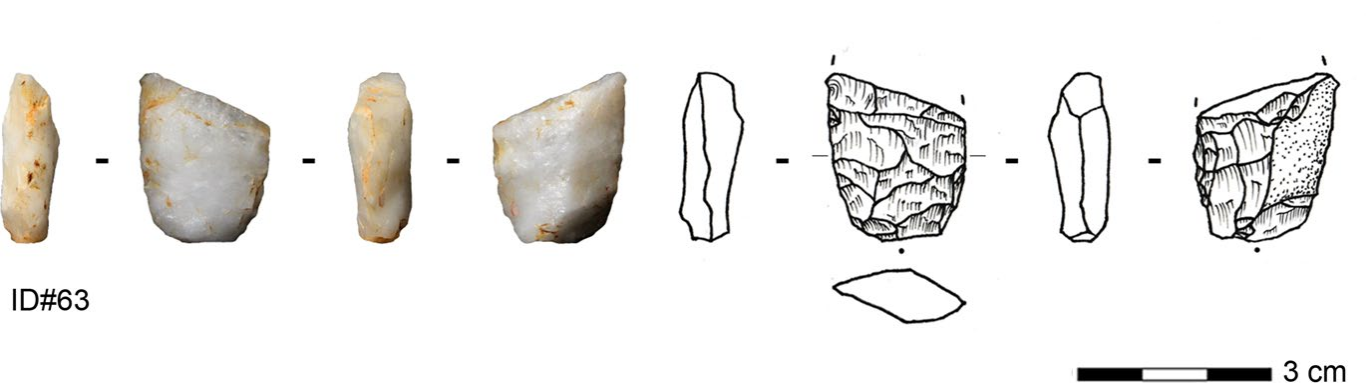
Bifacial point made on quartz from Toumboura III. The piece features manufacturing phase 2 (photo by V. C. Schmid; drawing by H. Würschem)

A great proportion of bifacial tools made on silexite and quartz pieces (*n* = 17) qualify for classification as bifacial points due to their regularity and symmetry (Figs. 7 and 8). Only one product is completely preserved, and most of the fragments sustained snap fractures at different locations. The latter often occurred as knapping errors during production. Finished points are most frequent (*n* = 9) (Fig. 7.2-5), followed by pieces from advanced shaping (*n* = 6; Figs. 7.1 and 8) and initial shaping (*n* = 2). Flakes and small slabs served as original blanks. A piece exhibiting 60% of cortex coverage and natural surfaces broke laterally during one of the first shaping operations. This piece, preserving the morphology of the small slab, provides information about the selected initial volume and has a preserved length of 44.3 mm and thickness of 14.5 mm. The start of the shaping involved hard hammer percussion, attested by deep concave negative bulbs of the removals.

In the advanced phase, knappers introduced soft hammer percussion and initiated the process of regularization of the outlines and the bifacial and bilateral symmetries. The complete piece belonging to phase 2a is 30-mm long and weighs 4.9 g. The mean width lies at 17.8 mm (median = 16.9 mm) and the mean thickness at 7.5 mm (median = 7.7 mm). The tools of the final phase show a mean maximum width of 12 mm (median = 12.1 mm) and a mean maximum thickness of 4.7 mm (median = 5 mm) (Table 10). These bifacials exhibit fairly narrow fusiform lanceolate morphologies. They have predominantly biconvex cross sections, showing that the toolmakers manufactured the bifacial points by alternating removals. They worked the two surfaces simultaneously, which implies the transformation of the entire volume to obtain and maintain the desired tool (Boëda, 1997, 2001; Nicoud, 2011).

**Table 10:** Descriptive statistics of width and thickness of different manufacturing phases of bifacial points at Toumboura III

	Width (mm)			Thickness (mm)		
	*Early shaping*	*Advanced shaping*	*Final shaping*	*Early shaping*	*Advanced shaping*	*Final shaping*
n	0	4	7	2	6	9
Min	-	13.9	8,7	6.3	3.0	2.3
Max	-	23.5	15.2	14.5	12.1	7.1
Sum	-	71.1	84.0	20.8	45.2	42.7
Mean	-	17.8	12.0	10.4	7.5	4.7
Std. error	-	2.2	0.8	4.1	1.2	0.6
SD	-	4.5	2.2	5.8	3.0	1,7
Median	-	16.9	12.1	10.4	7.7	5,0

The outstanding feature on these bifacial points lies in identifying subparallel and rectilinear shaping scars (e.g., Fig. 7.3), suggesting the use of pressure flaking, at least in the final shaping phase. Moreover, one bifacial point shows a removal scar from the right edge obliquely crossing the entire convexity of the surface to overpass the left edge (Fig. 7.2), unquestionably indicating the application of pressure technique on this category of bifacial tool (Inizan et al., 1999; Soriano & Huysecom, 2012). The majority of the bases are broken off, but the tips, with a regular pointed outline, have tip penetrating angles of 46° (median = 45°) and tip angles of 37° (median = 46°). The tip width ranges between 7.7 and 12 mm, and the tip thickness is 2.3–5.5 mm. The intermediate parts are slightly convergent and have mean edge angles of 52.2° (median = 48°). The bifacial points encompass a distal active, intermediate receptive, and proximal passive part. Due to the structure and morphometrics of these bifacials, they seem to be envisaged for axially hafted armatures, but we observed no impact fractures.

#### Bifacial Pieces with a Natural Back

**Fig. 9:**
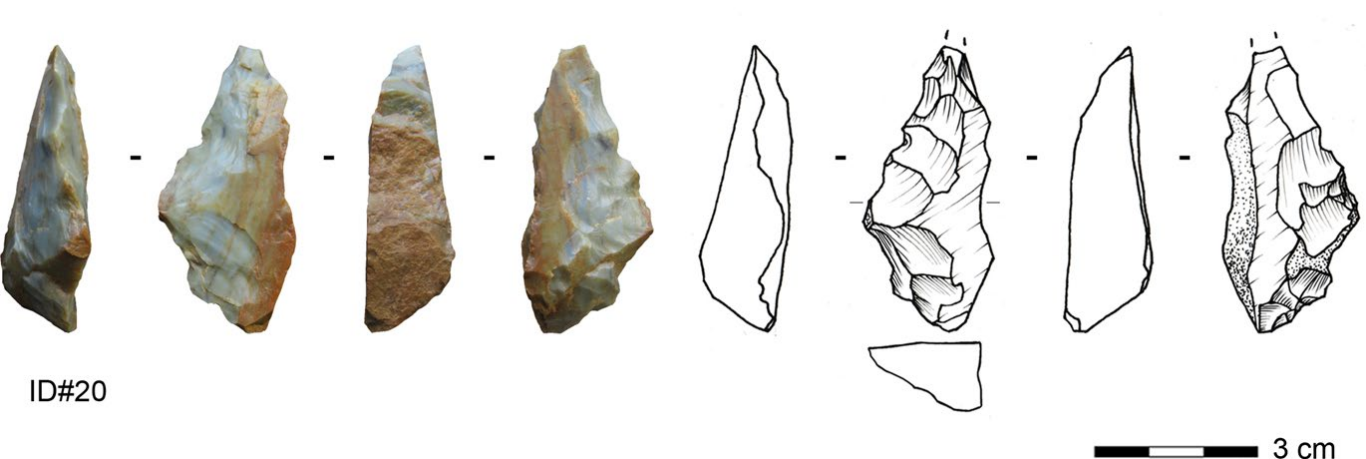
Bifacially backed knife made on silexite from Toumboura III (photo by V. C. Schmid; drawing by H. Würschem)

Four bifacial pieces have a natural back opposite to a robust working edge. One tool is made on sandstone, while the others are made on silexite (Fig. 9). Two of these are complete; one has a length of 36 mm and the other a length of 53.9 mm. The mean width is 19.7 mm (median = 19.2 mm), and the mean thickness is 11.4 mm (median = 11.7 mm). Two of the pieces attest that slabs were used as original blanks. The bifacial knives show exclusively planoconvex cross sections. In general, the distal parts exhibit a tip of a rather convex outline lacking a regular pointed outline. The bases are elliptic or triangular with an irregular outline. The organization of the shaping seems to have proceeded hierarchically. The knappers started to shape the flat surface of the blank with large removals and subsequently modified the working edge on the convex surface, causing the asymmetric section and allowing for frequent resharpening episodes.

The toolmakers shaped these tools asymmetrically in section and plain view to create a robust working edge opposite a natural back. The outline of the lateral opposite to the back is not continuously convex; instead, the delineation of the shaping demonstrates a break, like a half diamond. The section and angles of the shaping change from the distal towards the proximal part, possibly implying that the proximal part of this worked edge belonged to the prehensile part as well, in addition to the natural back.

#### General Presentation of the Shaping Flakes

The shaping flakes are mainly made on sandstone. The following results principally concern this rock type and, by extension, the bifacial pieces with planoconvex cross sections that are also mainly made on sandstone. Correlations could be established between the large spectrum of shaping flakes and the different phases of the manufacturing process of bifacial tools, from initial shaping to the finished tool, including resharpening and reconfiguration events. The phases identified for the shaping flakes are the (1) early shaping (1), two phases of advanced shaping (2a, 2b), the final shaping (3), and intermediate phases of restructuration (R).

#### Shaping Flakes from the Early Shaping Phase (1)

The early shaping phase is documented by 26% of the shaping blanks (*n* = 278; Fig. 10.1-3). The majority exhibits cortex coverage on their dorsal face (88.5%), including 32 completely cortical flakes, suggesting that the production of at least some bifacial tools started directly onsite with little preceding raw material roughout. The primary cortex types are weathered or have natural surfaces. Moreover, twelve of the 32 blanks without cortex are Kombewa flakes. This confirms that, in addition to natural raw material volumes, the toolmakers also selected large flakes to transform into bifacials.

**Fig. 10:**
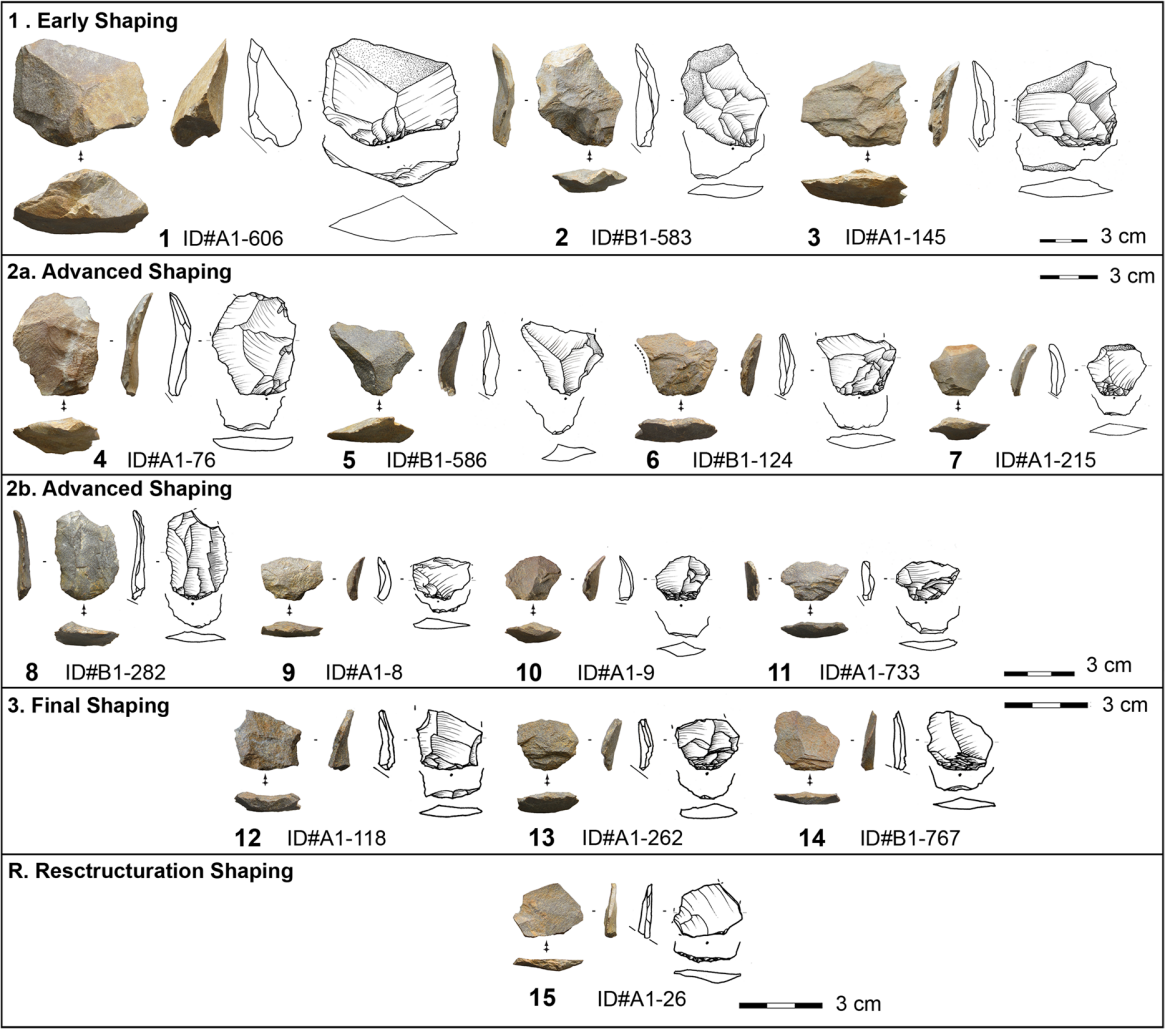
Shaping flakes from Toumboura III according to their phases of production. All made on sandstone. (photos by V. C. Schmid; drawings by H. Würschem)

Besides cortical and Kombewa flakes, some of the shaping flakes demonstrate technical features complying with products with *débordants* edges and products with large *débordants* platforms (*dos limités*). The products predominantly have trapezoidal or rectangular morphologies. Most of the flakes, 37.8%, show a rectilinear profile, but pieces with slightly curved and curved profiles are also common. About 74.1% of these shaping flakes exhibit two or fewer dorsal scars, and the scars are usually unidirectionally oriented.

Due to the incorporation of both slabs and flakes, diagnostic technical flake types are the outcome of the early shaping phase. The toolmakers needed to bevel one edge of the chosen slabs at the start to enable a subsequent bifacial transformation of the preform (see Fig. 5.1). To achieve the bevelling of the edge, they detached products with *débordants* edges and products with large *débordant* platforms from the slab corner, on the slab edge or the slab surface (Richter, 1997, p. 185ff.). The first of these products have cortical *débordants* edges, while pieces later in the bevelling operation have a *débordant* edge formed by large removals. Kombewa flakes result specifically from the shaping of the ventral face of flakes.

**Fig. 11:**
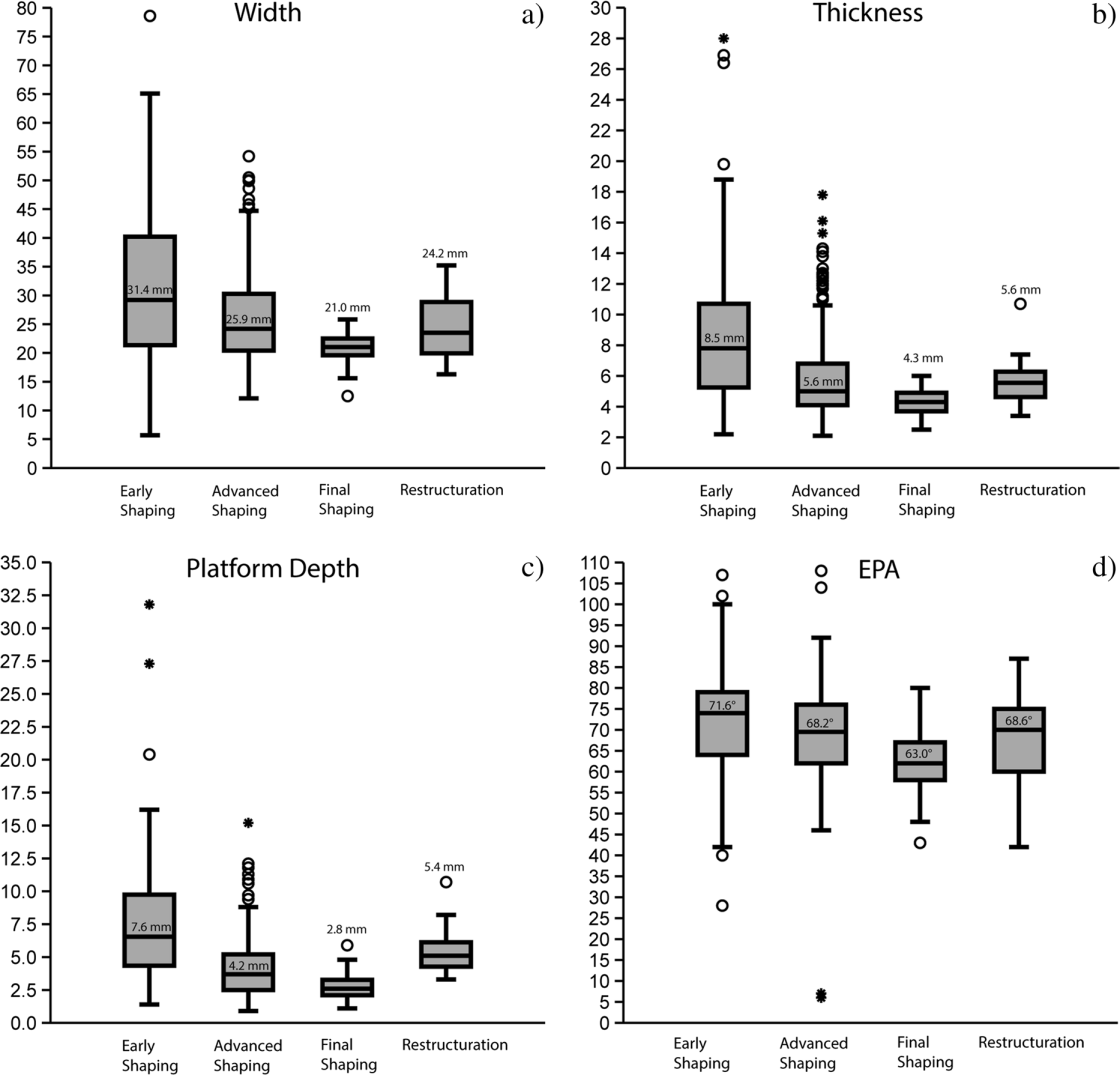
Dimensions of shaping flakes from Toumboura III. (a) Box plots of the width of shaping flakes in mm; (b) box plots of the thickness of shaping flakes in mm; (c) Box plots of platform depth of shaping flakes in mm; (d) box plots of external platform angle (EPA) of shaping flakes in degrees

The early shaping phase flakes are significantly larger than those of the subsequent phases (Fig. 11; SI 5). Most platforms are plain or cortical. The platform of one piece consists of parts of an old platform and ventral face coming from the flake that was shaped. Concerning the knapping technique, the several millimeter-thick platforms indicate internal percussion, i.e., striking further from the core margin. At the same time, dorsal reduction in the form of short, hinged removals implies an occasional marginal gesture, i.e., striking close to the core margin. The high frequency of lips on 66.4% of the completely and proximally preserved flakes attests tangential as the main motion. In addition to what was observed on the first phase bifacial tools, hard and soft stone hammers were used in the early shaping phase. The presence of Siret breaks and shattered bulbs further supports this observation.

#### Shaping Flakes from the Two Phases of Advanced Shaping (2a and 2b)

Advanced shaping constitutes the largest part of the shaping flakes, at 63.8% (*n* = 683) (Fig. 10.4-11). A total of 11.1% exhibit cortex on < 50% of dorsal coverage and mainly in the distal and lateral area or as natural back and 5.3% have cortical platforms. They are all from phase 2a. This phase belongs to advanced shaping and includes some Kombewa, *débordants*, and *dos limités* shaping flakes. The majority of the products (69%), especially the ones from the later advanced shaping, have slightly curved, curved, or highly curved profiles. The morphology is commonly trapezoidal or rectangular, and the dorsal scar patterns generally encompass two or more scars. The directions are primarily bidirectional (42.8%), followed by unidirectional (27.2%) and orthogonal (24.3%).

The advanced shaping flakes are significantly smaller than those of the early shaping phase and significantly larger than those of the final shaping phase (3) (Fig. 11; SI 5). Most platforms are plain (46.7%), while prepared platforms (faceted, dihedral, and *en éperon*) appear frequently (34.7%), and crushed platforms are also present (13.2%). The significantly smaller platform depth values (compared to those of the early shaping blanks) and the high frequency of external platform margin preparation (particularly of the advanced shaping flakes 2b) point to the importance of marginal percussion. The largest proportion of the completely and proximally preserved flakes (86.2%) have lips indicating a tangential knapping motion. The low occurrence of Siret breaks, shattered bulbs and pronounced bulbs, and the low EPA values (mean = 68.2°), especially among the later advanced shaping flakes, suggest a diminished use of stone hammers and increased use of soft organic percussion.

#### Shaping Flakes from the Final Shaping Phase (3)

Shaping flakes, identified as the result of the final shaping phase, make up only 7.2% (*n* = 77) (Fig. 10.12-14). However, a great proportion of flakes from this phase probably fall into the small fraction causing this biased underrepresentation. The products show primarily a trapezoidal morphology and a slightly to highly curved profile. The dorsal scar patterns generally encompass two or more scars, and the scars are exclusively unidirectional. The final shaping flakes are significantly smaller in size than those of the preceding phases (Fig. 11; SI-5). The majority of the pieces exhibit either prepared platforms (44.2%) or plain platforms (39%). The significantly smaller platform depth (mean = 2.8 mm) and EPA (mean = 63°) values of the flakes from the final shaping phase as compared to those of the early and advanced shaping blanks, the high proportion of dorsal reduction (95.3%), the presence of a lip (96.9%), and poorly developed or absent bulbs (95.3%) make a strong case for marginal, tangential soft organic percussion.

#### Shaping Flakes from the Intermediate Restructuration Phases (R)

Restructuration shaping flakes (*n* = 32) are not specific to a phase in the shaping sequence but are predominantly associated with later manufacturing phases (Fig. 10.15). When toolmakers desired to reconfigure their tool for production or functional reasons, they needed to create a platform to enable further knapping actions of resharpening and rectification of the working edge angles. The installation of the platform resulted in diagnostic restructuration products. The morphology is exclusively trapezoidal, and the profiles tend to be more rectilinear. The dorsal scar numbers range between one to four, and the dorsal scar pattern is usually unidirectional.

Regarding size, the restructuration flakes seem to occur in-between the advanced and final shaping blanks (Fig. 11; SI 5). All restructuration products have prepared striking platforms, and most of the platforms at 90.3% are faceted. The platform depth (mean = 5.4 mm) and EPA (mean = 68.6°) values, in addition to the absence of dorsal reduction, the appearance of shattered bulbs, and the highest frequency of lipping point to the use of internal, tangential soft stone hammer percussion. The dorsal face of these blanks comes from the flat face of the bifacial pieces with planoconvex cross section and is related to the large unidirectional removals on their plane surface. The striking platform of these specific shaping flakes is formed by the removals that caused an excessive steepness, impeding further resharpening, and, thus, commonly shows faceting. These blanks are detached with internal, tangential soft stone hammer percussion, and involve placing the knapping instrument on the convexly shaped surface as a platform and removing part of the flat surface. With this action, toolmakers attained a well-prepared platform to reconfigure the structure of the tool and to align the edge angle.

### Other Formal Tools

In contrast to the bifacials, the retouched elements (*n* = 126) exhibit less invasive modifications and are confined to the edges. The only exception is a single distal fragment of a unifacial point made on sandstone (Fig. 12.6). In addition to a thinning in the tip area, the tool shows a marginal dextrolateral ventral retouching. The piece has a lenticular cross section and a rectilinear profile. Generally, most of the other formal tools are made on sandstone (86.5%) and, to a lesser extent, on silexite (11.9%). The majority of the retouched pieces are fragmented. Toolmakers selected significantly large-sized blanks to modify them into tools (Table 11). More than half of the blank types used for tool manufacture involve shaping flakes (57.9%) and preferentially from early and advanced phases.

**Fig. 12:**
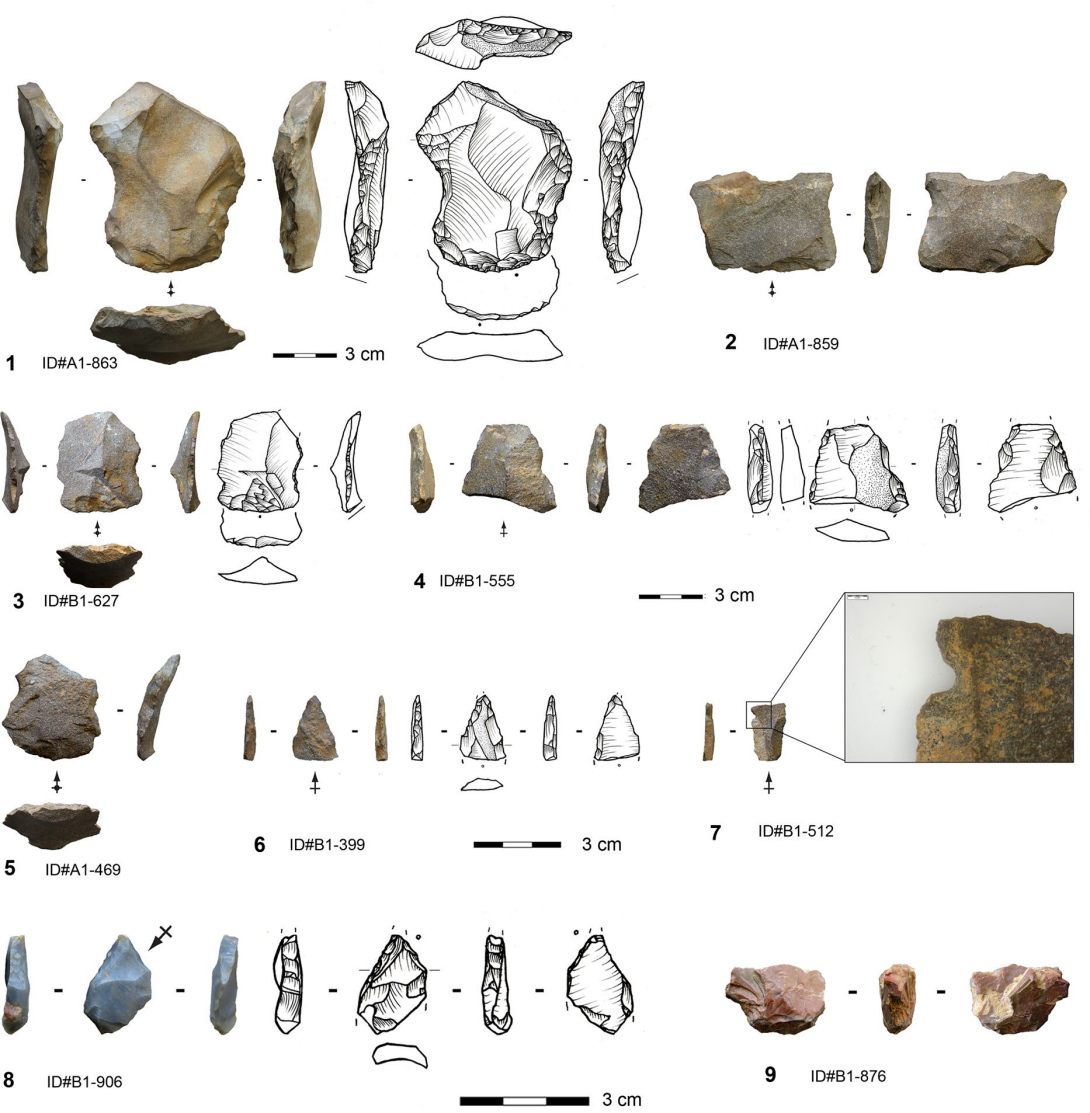
Formal tools from Toumboura III. 1 and 4 Multi-side scraper; 2–3 and 5 side scraper; 6 unifacial point with ventral thinning; 7 notched piece; 8 bec; 9 *pièce esquillée* (splintered piece). 1–7 Sandstone tools; 8 and 9 silexite tools (photos: by V.C. Schmid; drawings: by H. Würschem)

Side scrapers account for the largest number of formal tools (Fig. 12.2, 12.3, 12.5 and Table 4), and semi-abrupt, scaled retouch also occurs frequently on the ventral face (Fig. 12.2). The delineation of these tools is rather rectilinear and more concave than convex. Seven side scrapers are complete, showing a range of three to ten retouch removals forming their working edge. Notched pieces and denticulates are also common in the assemblage. Among those, a total of 10 notches show a pronounced concaveness indicative of the knapper’s clear control of the contact point and applied force (Fig. 12.7). This pattern in the notching could only have been achieved by pressure technique since other techniques, such as direct hammer percussion, would have involved much larger and shallower notches due to a larger contact area of the percussor on the worked edge. We also identified in the assemblage one notch resharpening flake made on sandstone, attesting to the reinstallation or indentation of a notch. The morphology of this flake is consistent with the application of pressure to make the notches.

**Table 11:** Descriptive statistics of length, width, and thickness of tools at Toumboura III (bifacial pieces not included)

	Length (mm)	Width (mm)	Thickness (mm)
*n*	22	58	125
Min	6.8	11.0	2.9
Max	86.7	68.2	23.5
Sum	802.0	1,926.7	927.4
Mean	36.5	33.2	7.4
Std. error	3.9	1.8	0.3
SD	18.4	13.6	3.2
Median	32.1	32.4	6.7
Mann–Whitney *U* test (tools to blanks)	U = 757.5, ***p* = 0.0079781**	U = 13,207, ***p* < 0.0001**	U = 72,437, ***p* < 0.0001**

Some tools also have multiple independent retouch locations, resulting in multi-side scrapers, multi-notched pieces, or composite tools (Fig. 12.1 and 12.4). Except for one medial-lateral fragment, all these tool types are made on early and advanced phase shaping flakes of great size and robusticity. In addition, the tool spectrum encompasses eight highly reduced *pièces esquillées* (splintered pieces). These artifacts are defined as quadrangular pieces with (sometimes bifacial) splintering, usually on two opposite ends, that are not related to retouching but to percussion or use (Hayden, 1980; Le Brun-Ricalens, 2006). Half of these are made on sandstone and the other half on silexite (Fig. 12.9). Furthermore, four end scrapers occur in the assemblage, from which two are exhausted to the extent that their *fronts* (end scraper caps) are flattened. Finally, two *becs* made on sandstone and silexite are part of the tool corpus. They have protruding working edges not in the form of long drill bits but shorter and with a sharp tip not clearly separated from the body. The angles of the intensively retouched working edges forming the tips of both pieces are abrupt and over 70° (Fig. 12.8), corresponding to a partial backing on the distal end (Tixier, 1974). This partial backing cannot be equated with the backing applied to create backed points and segments, a phenomenon known in West Africa only from MIS 2 onwards (Chevrier et al., 2016, 2018). The tips of both objects slightly broke off, probably during use.

The investment in standardization of side scrapers, notched pieces, denticulates, and combination tools is relatively low compared to the bifacials. Toolmakers seem to have pursued a strategy of choosing blanks from the supply of shaping flakes for immediate tool manufacture and use, with occasional resharpening or reconfiguration events. However, pressure flaking seems to have been implemented for both bifacial tools and notches for different purposes, a point that is further developed in the discussion below.

## Discussion

### The MIS 3 Middle Stone Age Lithic Assemblage of TMB III

This extensive study of the MSA lithic materials of TMB III, dating to between ca. 40 ka and 30 ka, emphasizes behavioral patterns that are yet unparalleled in West Africa for this period. Besides the findings on the raw material economy, the analysis illustrates an overwhelming dominance of bifacial tool production. Another outstanding feature of this assemblage is the use of pressure flaking for making bifacials and some of the notched pieces.

Several knapping techniques used for shaping the bifacial tools provide insights on the operational sequence of the knapping, such as gestures and choices made by the knappers to conform to their production objectives. These techniques are:

1. Mainly direct hard hammer percussion, tangential, internal, or marginal in the early shaping
2. Direct hard and soft hammer percussion for the advanced phase of manufacture
3. Direct soft hammer percussion exclusively in final and restructuration phases
4. Pressure flaking in the final shaping of bifacial points made on silexite

We consequently claim that the toolmakers of TMB III had great flexibility in problem-solving during stone knapping, highlighting a high level of adaptation and broad technical knowledge and understanding of rock properties. We elaborate further on the use of pressure flaking in the following section.

The raw material and morphometrics showed that two main types of bifacials were produced following adapted shaping procedures within the operational scheme, and probably infer differences in function.

The first group comprises large bifacial pieces made on sandstone and quartzite, with planoconvex sections, and morphology in plain view ranging from pointed oval to elongated narrow with parallel edges. These show a hierarchical bifacial tool production starting on the flat surface and continuing on the convex surface of the original blanks. The hierarchical production allows for a potentially long life cycle through multiple resharpening events that do not lead to structural changes. These bifacial tools are interpreted as rather suitable for cutting actions. The second group features small (ca. < 50 mm) and standardized bifacial tools with pointed ends, made on silexite and quartz (with biconvex sections), and fusiform lanceolate shapes in plain view. The shaping of the volume was achieved with alternating removals. These small bifacial points seem to be suitable for penetrating motions and could potentially be used as axially hafted armatures.

Beyond the possible raw material constraints which could have led to the production of two different bifacial tool types, we interpret this dichotomy as the indication that the bifacials at TMB III are not a one-tool-fits-all type. Their production suggests goal-directed actions. The shaping process is implemented following at least two different concepts: “bifacial as tool” (i.e., bifacial points) versus “bifacial used as blank for tool production” (i.e., bifacials with planoconvex cross section and bifacials with a natural back) (Boëda, 1997; Nicoud, 2011). The knappers may also have factored in the specific properties of the different raw materials: selecting sandstone for its toughness, rigidness, durable edges, and long life spans and silexite as high-quality raw material, in terms of high technical requirements, for the production of armatures and use of pressure flaking.

Our integrative techno-economic approach on all lithic components shows that more than half of the retouched pieces (58%), including primarily side scrapers, notched pieces, and denticulate, are made on early and advanced shaping flakes rather than flakes obtained by core reduction. At TMB III, shaping flakes are not necessarily waste products but are intertwined for further usage in the larger techno-economic system (Claud, 2015; Faivre, 2003). However, by customizing the retouch position to the geometry of the tool blanks and the frequent utilization of multiple retouch locations on one piece, it seems that these tools – side scrapers and notched pieces – were meant for immediate use (in contrast to the bifacials).

The importance of bifacial shaping for the production of bifacial tools and blanks for other types of tools is corroborated by the minor investment in core reduction. Besides the cores being scarce, their exploitation (algorithmic) is simple and rather expedient for circumstantial purposes. The provenance of the large flakes used as blanks for the large bifacials and other tool groups remains open. They are not produced onsite, as no associated products, such as cores and preparation or maintenance flakes, are present. They were probably introduced to the site in the same way as the natural slabs. To the contrary, bifacial pieces are manufactured in situ and occasionally transported away from the site (sandstone), fabricated for export, and left on site when broken (silexite), or brought to the site for utilization, resharpening, and finally discard (quartzite). All of these show the employment of a mix of provisioning strategies between place provisioning (the exploitation of exclusively local rocks and presence of complete shaping sequences for sandstone and silexite) and individual provisioning (the import, use, resharpening, and discard of finished long-lasting quartzite bifacials) (Kuhn, 1995, 2004). Considering these elements, the site use seems to conform to a residential or short-term occupation, where stone knapping was an important activity.

### Implications of the Bifacial Shaping at TMB III

Bifacial technology is in itself probably one of the most reinvented and widespread technologies of past stone-knapping societies, and it is common within numerous industries in the African MSA. However, it occurs in different proportions, shapes, and morphometrics (e.g., McBrearty & Brooks, 2000). The cultural influences or traditional meanings behind its recurrent occurrence need to be demonstrated and delimited in the contexts of technological and environmental constraints (Otte, 2003). Nevertheless, some parallels can be drawn between the evidence of bifacial shaping at TMB III and other MSA contexts.

As highlighted above, one of the specificities of the assemblage of TMB III is the overwhelming dominance of bifacial shaping and production of bifacial pieces in the lithic technical system. This kind of technical system is well described for one other assemblage in Africa, namely the Still Bay of Sibudu Cave, with an age of 70.5 ± 2.4 ka (Soriano et al., 2009, 2015). In this site, shaping by-products account for 92% of the total lithic artifacts, and bifacially shaped elements form 53% of the tool corpus. The Still Bay bifacial points conform to the bifacial pieces with planoconvex cross sections at TMB III, probably intended to function primarily as pointed cutting devices with prolonged use (Soriano et al., 2015). The discrepancy in time and distance between these two MSA sites renders a common origin from the same technological tradition unlikely. We would rather claim a behavioral convergence in mobility strategies, territorial organization, and long-term planning that led to a similar pattern in emphasizing bifacial shaping. The hunter-gatherers in both places took advantage of resharpenable specialized bifacial implements for different purposes, and having at hand a ready supply of flakes when moving around in the landscape was a priority (see Hayden, 1979; Kelly, 1988; Soressi, 2004; Thomson, 1964). However, the TMB III assemblage is distinguished from the Still Bay by lacking a technological, morphometric, raw material-related – and probably functional – dichotomy among bifacial pieces.

The dichotomy between types of bifacial pieces, an outstanding characteristic of the assemblage of TMB III, does not find many parallels. However, the dichotomy between different point types within MSA assemblages is more frequently reported (see for overview Douze et al., 2021). The example of the North-African Aterian is noteworthy in this context since the bifacial component of the Aterian, and the establishment of adapted shaping procedures to gain different convergent tools is similar to the TMB III assemblage. The distinctive marker of the Aterian, which ranges from MIS 6 to MIS 3, is the presence of pedunculate or tanged tools and bifacial foliates (Bouzouggar & Barton, 2012; Hawkins, 2001; Scerri, 2017; Tixier, 1967). The Aterian covers a large geographic area from the Maghreb to the Western Desert of Egypt and the Sahel region (Débenath et al., 1986; Scerri, 2017; Spinapolice & Garcea, 2013; Tomasso & Rots, 2018; van Peer, 2001; Wendorf & Schild, 1992). The pedunculates in Senegal have been interpreted as possible markers of northern influences (Niang et al., 2018; Scerri et al., 2016). As at TMB III, the bifacial foliates of the Aterian appear as a complementary tool type. Indeed, the morphological differences and the particularity of the tang arrangement advocate that their place in the technological system was unlikely interchangeable with the bifacial foliates and that two manufacturing sequences were pursued (Spinapolice & Garcea, 2014). However, the affinities cannot be taken further because shaping is of minor importance in the Aterian where Levallois methods, discoid reduction strategies, and blade production predominate (Bouzouggar & Barton, 2012; Scerri, 2017; Spinapolice & Garcea, 2013).

### Implications of Pressure Flaking at TMB III

Compared to the temporally and spatially recurrent bifacial shaping, the situation differs when it comes to the application of pressure flaking. This flaking method was used at TMB III for standardization and precise thinning in the final shaping phase of small bifacial points made on silexite (*n* = 9) and for the notching of some of the notched pieces, denticulates, and multi-notched pieces (*n* = 14/40). The pronounced concaveness of the notches attests to the application of pressure notching. These MIS 3 MSA knappers, therefore, mastered the application of pressure technique to achieve versatility in lithic production (e.g., notching and retouching) and to create different tool types (e.g., notched pieces and bifacial points).

The earliest examples of pressure technique were documented in the South African MSA at Sibudu Cave D-A layers (Rots et al., 2017), Umhlatuzana Rockshelter (Högberg & Lombard, 2016), Blombos Cave (Mourre et al., 2010), and purportedly Hollow Rockshelter (Högberg & Larsson, 2011), all older than 70 ka but falling within the Upper Pleistocene. Later occurrences are also described in the Howiesons Poort layers of Sibudu, dated to ca. 65–62 ka, which encompass bone compressors and quartz bifacial points, indicating the use of pressure flaking (de la Peña et al., 2013; d'Errico et al., 2012). In the older D-A layers at Sibudu, the pressure technique was used to produce serrated bifacial points. The assemblage also contains a larger number of bifacial pieces without the application of pressure. The serrations determined the final stage of the pieces, as no reworking was observed and seemingly served a functional objective distinct from the remaining bifacials without serrations. At TMB III, as in the D-A layers at Sibudu, pressure technique is linked to a particular shaping sequence within the operational scheme resulting in specific tool types. In the case of the D-A layers at Sibudu, toolmakers applied pressure notching to serrate the edges, while at TMB III, pressure flaking was applied to regularize the edges and thin the pieces. In contrast to TMB III, where pressure was also used for creating notches on non-bifacial tools, the notched pieces and denticulates in the D-A layers were consistently retouched by percussion. In another context, the application of pressure flaking on Still Bay points at Blombos seems to be connected with a final shaping phase of bifacials’ tips after heat treatment. It is interesting to mention that, as for TMB III, bifacial shaping is central to Still Bay assemblages, which raises the question of the (re-)invention of pressure flaking in the context of technical systems that emphasized bifacial shaping.

At Aterian sites in Jebel Gharbi, northwestern Libya, ca. 43–44 ka, the tang production of pedunculates involved two different techniques depending on the raw material (Garcea, 2012). While knappers created the tangs of the quartzite points with direct hard percussion and subsequent perpendicular abrasion, they applied pressure flaking with either a small hammerstone or a soft hammer, such as antler, to form the flint tangs (Garcea, 2012). Concordance with TMB III exists in the association of pressure technique with a specific raw material, but there are differences in the purpose of this technique. Outside of Africa, the pressure technique appeared much later. It is observed in relation to the laurel leaf manufacture of the European Solutrean techno-complex with an age range of ca. 21.5–17 ka (Aubry et al., 2008; Schmidt & Morala, 2019; Smith, 1966; Walter et al., 2013); the fluted projectile point production of the Terminal Pleistocene Paleoindian period of North, Central, and South America (Acosta-Ochoa et al., 2019; Nami, 2021; O’Brien et al., 2014); the bifacial projectile point manufacture of the Arabian Neolithic (Charpentier et al., 2002; Crassard et al., 2020); and, finally, the fabrication of Kimberley points around 1.4 ka BP in the Kimberley Region of Northwest Australia (Akerman & Bindon, 1995; Akerman et al., 2002; Harrison, 2004; Moore, 2015).

In West Africa, the HA1 Neolithic assemblage from Ravin de la Mouche within the Ounjougou site complex, dated to the first half of the 10th-millennium cal BC, contains small foliate, planoconvex bifacial points classified as arrowheads and finalized by pressure technique (Soriano & Huysecom, 2012). TMB III thus substantiates the appearance of this distinctive invention in the manufacturing sequence for bifacial points with particular morphological features as the oldest evidence in West Africa and early in the broad African context.

### TMB III Within the MSA Context of West Africa and Cultural Dynamics During MIS 3

MIS 3 is the best-represented period in the West African MSA and allows for comparisons, especially with assemblages from the Ounjougou site complex in Mali and Tiémassas in Senegal. Here, we discuss possible scenarios of population dynamics, behavioral patterns of change, and technical novelties during this phase of the MSA. At the Ounjougou complex in Mali, Coupe Franck, Dandoli 1 and 3, and Ravin de la Vipère demonstrate that past human groups most frequently used the discoid reduction on quartz as well as sandstone and bipolar-on-anvil flaking of quartz cobbles (Chevrier et al., 2018; Soriano et al., 2011). At the site of Orosobo, the otherwise rarely appearing Levallois concept is found exclusively in the upper layer dated to ca. 30 ka and discoid together with centripetal recurrent Levallois methods occur in the lower layer with an age of ca. 40 ka (Robert et al., 1999). Additionally, a blade production on sandstone, unique in West Africa, was identified at Oumounaama Atelier , dating to around 60 ka. Finally, several sites, such as Oumounaama Px, Kondo, Songona 1, Dandoli Ouest, Draperies, and Oumounaama Butte, yielded evidence for the manufacture of bifacial pieces and foliate points, extending from early MIS 3 to the onset of MIS 2 (Chevrier et al., 2018). In contrast to the other sites with bifacial shaping, Songona 1 (ca. 40 ka) exhibits an algorithmic unidirectional reduction strategy for blank production, carried out on quartz (Huysecom et al., 2009).

In Senegal, the reanalyzed and reexcavated littoral site of Tiémassas comprises MSA lithic assemblages from three discrete stratified archaeological horizons of broadly homogeneous nature dating to 61.9 ± 2.6 ka, 47.3 ± 2.9 ka, and 25.9 ± 1.3 ka (Niang et al., 2020). The raw materials are sandstone and locally available flint. A range of reduction strategies, from preferential, centripetal, and unidirectional Levallois methods to the discoid concept and single or multi-platform blank production, seem to have originated predominantly from the upper two layers. The tool corpus consists mainly of different side scraper types with a small number showing bifacial retouching, limaces, denticulates, and notched pieces (Niang et al., 2018, 2020).

Although similarities with sites from Mali (especially Songona 1) can be observed, particularly concerning the presence of bifacial technology, TMB III lacks evidence of blank production that was based on discoid or Levallois methods. On the contrary, similar to the case of the blade production at Oumaounaama Atelier, the assemblage of TMB III seems to add to a restricted number of distinct industries identified during MIS 3 in West Africa. The prevalence of bifacial technology, the manufacturing of two different bifacial tool types following specific shaping strategies, and the application of pressure techniques remain the exclusive technological features of TMB III.

By contributing to the technological diversity within the late MSA of West Africa, TMB III reinforces the image of a complex occupational history in the region between 60 and 25 ka, during which most dated West African MSA sites occur. This image of techno-culturally heterogeneous groups, probably evolving in contrasting landscapes, raises the question of episodes of migration, contraction, fragmentation, and possible regional extinction of population groups as currently suggested in palaeogenetics (Schlebusch & Jakobsson, 2018; Skoglund et al., 2017).

Given these complex dynamics, the variety of technological solutions, concerning reduction schemes and tool manufacture, at different places and times is to be expected (see Richter, 2016). In turn, the manifestation of innovations, associated with social transmission and cumulative cultural evolution, depends equally on three demographic parameters: population density, group size, and the quality network system between populations (Henrich, 2004, 2015). In the case of the archaeological record known from the Falémé Valley, TMB III stands out alone. Therefore, it is premature to estimate the significance of the adoption of pressure flaking within an assemblage dominated by bifacial technology and to interpret the demographic parameters accompanying or respectively facilitating such innovative behavior. The renewed research on the West African Upper Pleistocene and early Holocene promises to bring clarity in the near future. 

## Conclusion

The days of marginalizing West Africa in the study of human cultural evolution have passed. Our study of the lithic assemblage of TMB III highlights the dominance of bifacial technology, the manufacture of distinct bifacial tool types, the exploitation of shaping flakes as blanks for tool production, and the application of pressure technique. These results enhance the knowledge on the diversity of technological phenomena and behavioral innovations associated with West African MSA during the MIS 3 period. The archaeological record points to a complex scenario of population dynamics, technological developments, and cultural change (Chevrier et al., 2016, 2018; Niang et al., 2020; Scerri et al., 2016, 2017). However, although the body of archaeological data has increased since the last decade in this region, it is still too early to make definitive inferences about the driving forces for these cultural innovations. Yet, TMB III provides the largest lithic assemblage from a well-dated and well-stratified West African context and demonstrates a specific technological expression that represents a benchmark for the settlement history and chrono-cultural framework of the MSA in West Africa. The ongoing “Human Population and Paleoenvironment in Africa” project in the Falémé Valley’s MIS 3 and 2 contexts promises to refine the MSA cultural trends and shed new light on the causes and mechanisms, such as demographic fluctuations or influences of traumatic climatic and environmental upheavals, behind MSA’s population history and technological changes in West Africa.

## Supplementary Information

Below is the link to the electronic supplementary material.

Supplementary file1 (DOCX 660 KB)
