# Supplementary material for: Middle Stone Age Bifacial Technology and Pressure Flaking at the MIS 3 Site of Toumboura III, Eastern Senegal
Source: Afr Archaeol Rev. 2021 Nov 25;39(1):1–33. doi: 10.1007/s10437-021-09463-5 (PMC9046311; doi:10.1007/s10437-021-09463-5)
Supplement: Supplementary file 1 — Supplementary file1 (DOCX 660 KB) [file 10437_2021_9463_MOESM1_ESM.docx]

**Supplementary Information**

**SI 1** Attribute list used for the analysis of the lithic assemblage at Toumboura III. (*for blanks following the definition of Auffermann, et al., 1990 and Nigst, 2012; for cores following the definition of Hahn, 1982 and Nigst, 2012)

| **General** | **Blanks** | **Tools** | **Cores** |
| --- | --- | --- | --- |
| Refitting affiliation | Blank type | Tool type | Core type |
| Raw material category | Diagnostic technical category | Position of retouch | Original morphology |
| Raw material unit | Morphology | Location of retouch | Number of striking platforms |
| Patination | Dorsal scar number | Delineation of retouch | Relation of platforms |
| Thermal alteration | Dorsal scar orientation | Extent of retouch | Number of removal surfaces |
| Cortex proportion | Platform type | Morphology of retouch | Orientation negatives on removal surface |
| Edge state | Platform form | Angle of retouch | Length of removal surface |
| Fragmentation | Platform width | Number of retouch scars | Width of removal surface |
| Technical accident | Platform depth | Fragmentation of retouch | Shape of removal surface |
| Length* | Dorsal reduction | Number of retouched edges | Striking platform type |
| Width* | Lipping |  | Angle between removal surface and striking platform |
| Thickness* | Hertzian cone |  | Last operation |
| Weight | Bulb |  | Last productional operation |
| Maximum dimension | Shattered bulb |  | Length of last end product |
|  | Bulbar scar |  | Width of last end product |
|  | Exterior platform angle |  | Core back |
|  | Interior platform angle |  | Core base |
|  | Distal termination |  | Stigmata of technique |
|  | Cross section |  | Discard factor |
|  | Profile |  |  |

**SI 2** Recording form with attributes especially compiled for the technological and techno-functional analysis of the bifacially pieces of Toumboura III


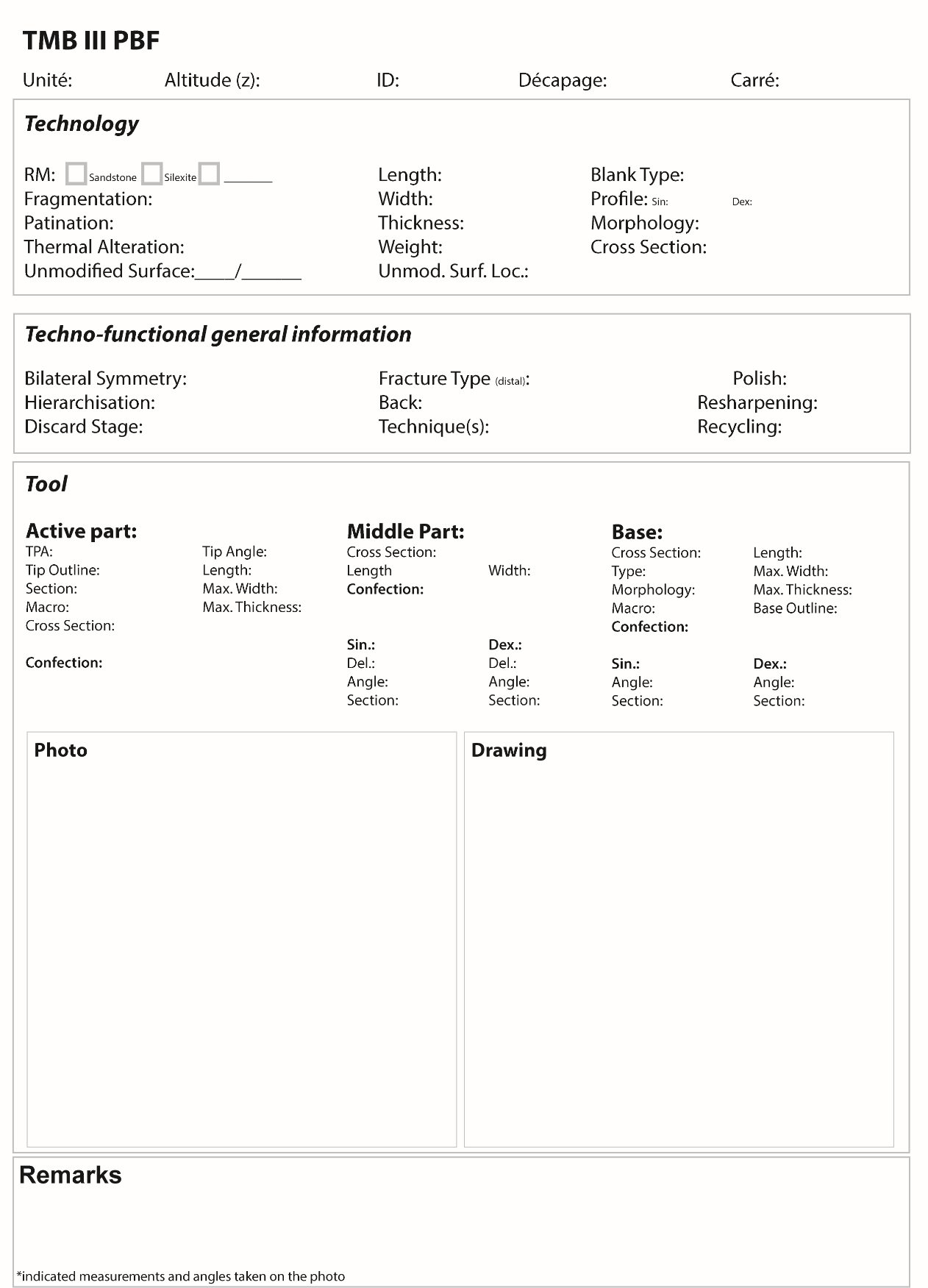


**SI 3** 3D models by photogrammetry, Toumboura III

The 3D models have been made by photogrammetry. For the shooting, we used a camera Canon EOS 6D, a lens Canon EF 100 mm 1 :2.8 L IS USM, an automatic turntable, a set of 12 bits coded targets, a tripod and a light box Havox HBP-40XD with 6 dimmable LED bar with a 93 CRI. The 3D models were generated with the software Agisoft Metashape v.1.6.2. A classical workflow was used for close-range photogrammetric modeling using the following parameters in Metashape: first, with the alignment in high accuracy of the set of photos; second, the building of a dense cloud in high quality; third, the building of a mesh in high quality, the scaling with 12 bits targets and then the building of the texture in the generic or the adaptive orthophoto mapping mode with a 4096 texture size/count.

Three archaeological pieces have been modeled in 3D. The 3D models can be found at the following address online:

- preform of a bifacial piece TMBIII_UJ_A1_22 => <https://visual.ariadne-infrastructure.eu/3d/d21a2b2d296789d71d1b62e8ba2eb0e9>


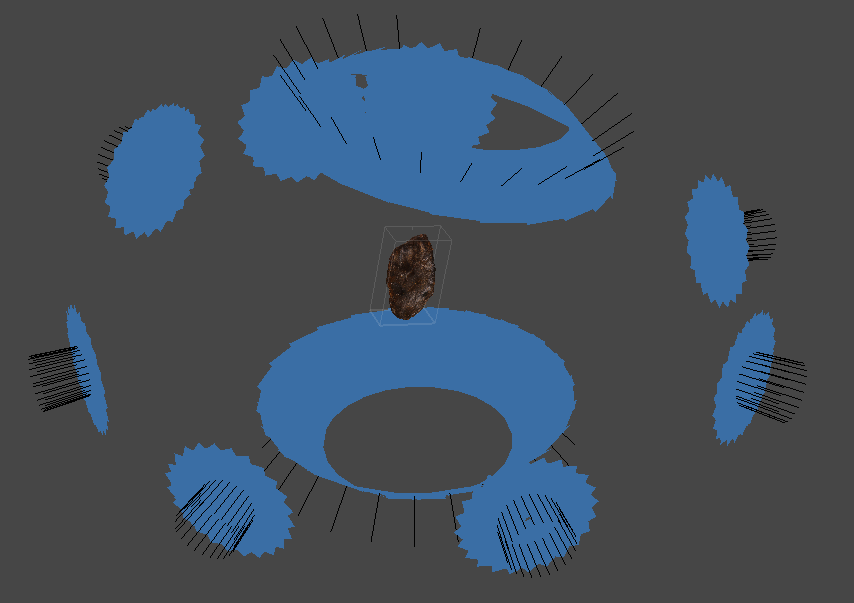


- bifacial piece TMBIII_UJ_A1_17 => <https://visual.ariadne-infrastructure.eu/3d/6b91c135583c27766a9fcbb18f7c7ed6>


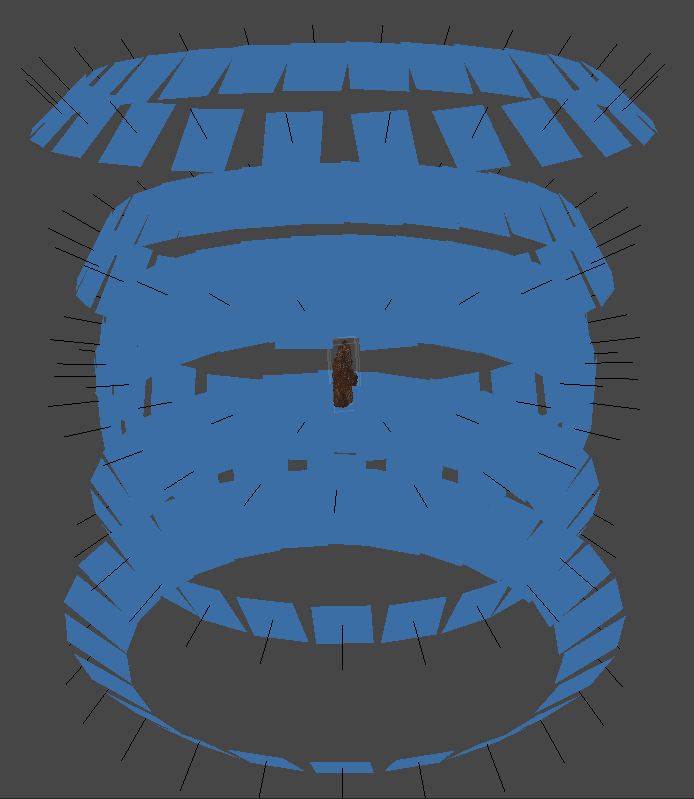


- bifacial piece TMBIII_UJ_A1_16 => <https://visual.ariadne-infrastructure.eu/3d/10de63c867503ea7413042de3d2dd12b>


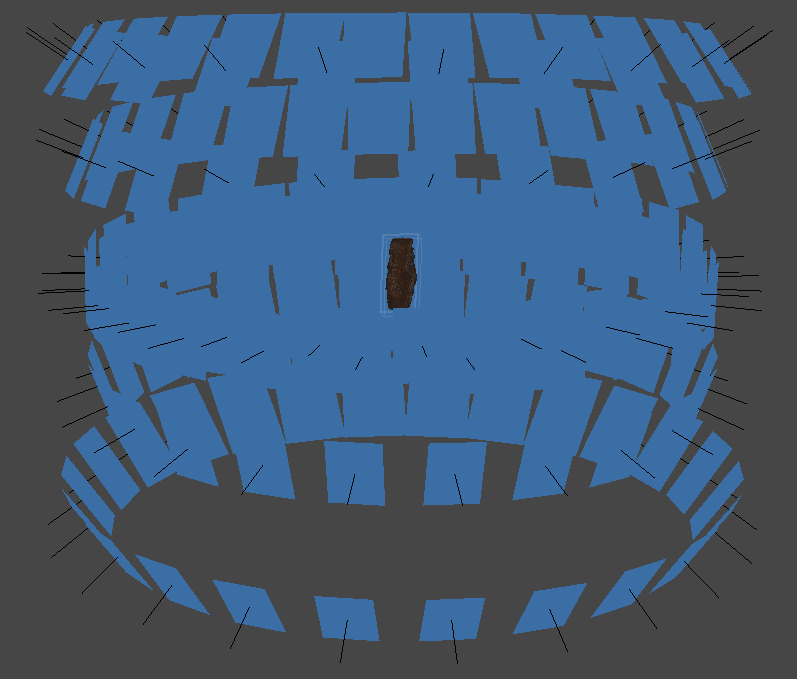


The detailed processing parameters and proprieties of the three 3D models are presented hereafter:

1) Processing Parameters for the 3D model of the preform of a bifacial piece TMBIII_UJ_A1_22

**General**

Cameras 239

Aligned cameras 239

Markers 30

Scale bars 14

Coordinate system Local Coordinates (m)

Rotation angles Yaw, Pitch, Roll

**Point Cloud**

Points 678,908 of 835,275

RMS reprojection error 0.189069 (0.834296 pix)

Max reprojection error 7.89878 (49.0869 pix)

Mean key point size 3.60873 pix

Point colors 3 bands, uint8

Key points No

Average tie point multiplicity 8.37882

**Alignment parameters**

Accuracy High

Generic preselection No

Reference preselection Estimated

Key point limit 400,000

Tie point limit 50,000

Filter points by mask Yes

Mask tie points No

Guided image matching Yes

Adaptive camera model fitting Yes

Matching time 7 hours 8 minutes

Matching memory usage 1.04 GB

Alignment time 31 minutes 51 seconds

Alignment memory usage 546.39 MB

**Optimization parameters**

Parameters f, b1, b2, cx, cy, k1-k4, p1, p2

Fit additional corrections Yes

Adaptive camera model fitting Yes

Optimization time 3 minutes 29 seconds

**Depth Maps**

Count 239

**Depth maps generation parameters**

Quality High

Filtering mode Aggressive

**Dense Point Cloud**

Points 9,880,354

Point colors 3 bands, uint8

Depth maps generation parameters

Quality High

Filtering mode Aggressive

Processing time 6 hours 15 minutes

**Dense cloud generation parameters**

Processing time 1 hours 51 minutes

**Model**

Faces 1,500,000

Vertices 750,224

Vertex colors 3 bands, uint8

Texture 4,096 x 4,096, 3 bands, uint8

**Texturing parameters**

Mapping mode Generic

UV mapping time 1 minutes 55 seconds

**System**

Software name Agisoft Metashape Professional

Software version 1.6.2 build 10247

OS Windows 64 bit

RAM 63.85 GB

CPU Intel(R) Xeon(R) CPU E3-1505M v6 @ 3.00GHz

GPU(s) Quadro M2200

2) Proprieties of the 3D model of the preform of a bifacial piece TMBIII_UJ_A1_22

**Scaling**

Label Distance (m) Error (m)

target 14_target 20 0.0897803 -0.000219704

target 15_target 21 0.0895815 -0.000418542

target 16_target 22 0.0896309 -0.000369117

target 15_target 21 0.0895639 -0.000436077

target 16_target 22 0.0896096 -0.000390404

target 14_target 20 0.0901806 0.000180557

target 15_target 21 0.0899829 -1.70578e-05

target 16_target 22 0.090019 1.90187e-05

target 17_target 23 0.0900778 7.78329e-05

target 18_target 24 0.0901832 0.000183195

target 14_target 20 0.0904567 0.000456696

target 15_target 21 0.0902035 0.000203463

target 17_target 23 0.0902937 0.000293678

target 18_target 24 0.0904222 0.000422157

Total 0.000303501

**Resolution:** 0.0956 mm/pix

**Point density:** 109 points/mm²


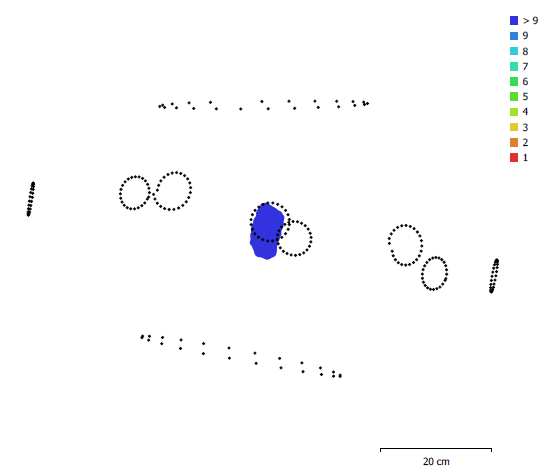


Fig. 1. Camera locations and image overlap of the 3D model of the preform of a bifacial piece TMBIII_UJ_A1_22.

3) Processing Parameters for the 3D model of the bifacial piece TMBIII_UJ_A1_17

**General**

Cameras 144

Aligned cameras 144

Markers 48

Scale bars 24

Coordinate system Local Coordinates (m)

Rotation angles Yaw, Pitch, Roll

**Point Cloud**

Points 220,247 of 267,713

RMS reprojection error 0.124631 (0.642417 pix)

Max reprojection error 0.498044 (34.0123 pix)

Mean key point size 3.96986 pix

Point colors 3 bands, uint8

Key points No

Average tie point multiplicity 2.97122

**Optimization parameters**

Parameters f, b1, b2, cx, cy, k1-k4, p1, p2

Adaptive camera model fitting Yes

Optimization time 6 seconds

**Depth Maps**

Count 144

**Depth maps generation parameters**

Quality High

Filtering mode Mild

Processing time 20 minutes 52 seconds

**Dense Point Cloud**

Points 5,097,445

Point colors 3 bands, uint8

**Depth maps generation parameters**

Quality High

Filtering mode Mild

Processing time 20 minutes 52 seconds

**Dense cloud generation parameters**

Processing time 21 minutes 39 seconds

**Model**

Faces 1,500,000

Vertices 750,002

Vertex colors 3 bands, uint8

Texture 4,096 x 4,096, 3 bands, uint8

**Depth maps generation parameters**

Quality High

Filtering mode Mild

Processing time 20 minutes 52 seconds

**Reconstruction parameters**

Surface type Arbitrary

Source data Dense cloud

Interpolation Enabled

Strict volumetric masks No

Processing time 3 minutes 49 seconds

**Texturing parameters**

Mapping mode Adaptive orthophoto

**UV mapping time 1 minutes 36 seconds**

**System**

Software name Agisoft Metashape Professional

Software version 1.6.2 build 10247

OS Windows 64 bit

RAM 63.85 GB

CPU Intel(R) Xeon(R) CPU E3-1505M v6 @ 3.00GHz

GPU(s) Quadro M2200

4) Proprieties of the 3D model of the bifacial piece TMBIII_UJ_A1_17

**Scaling**

Label Distance (m) Error (m)

target 1a_target 7a 0.045125 0.000125019

target 2a_target 8a 0.0451994 0.000199373

target 3a_target 9a 0.0451344 0.000134406

target 4a_target 10a 0.0450587 5.86679e-05

target 5a_target 11a 0.0450313 3.12711e-05

target 6a_target 12a 0.0450836 8.35893e-05

target 13a_target 19a 0.0904157 0.000415715

target 14a_target 20a 0.0904032 0.000403244

target 15a_target 21a 0.090201 0.000201025

target 16a_target 22a 0.0902375 0.000237535

target 17a_target 23a 0.0903285 0.000328475

target 18a_target 24a 0.0904194 0.000419413

target 1_target 7 0.0448457 -0.000154329

target 2_target 8 0.0449093 -9.07052e-05

target 3_target 9 0.0448479 -0.000152105

target 4_target 10 0.0447596 -0.000240361

target 5_target 11 0.0447226 -0.000277352

target 6_target 12 0.0447905 -0.000209486

target 13_target 19 0.089788 -0.000211957

target 14_target 20 0.0897805 -0.000219529

target 15_target 21 0.0895927 -0.000407294

target 16_target 22 0.0896041 -0.000395852

target 17_target 23 0.0896743 -0.000325701

target 18_target 24 0.0897831 -0.000216877

Total 0.000258481

**Resolution:** 0.0389 mm/pix

**Point density:** 661 points/mm²


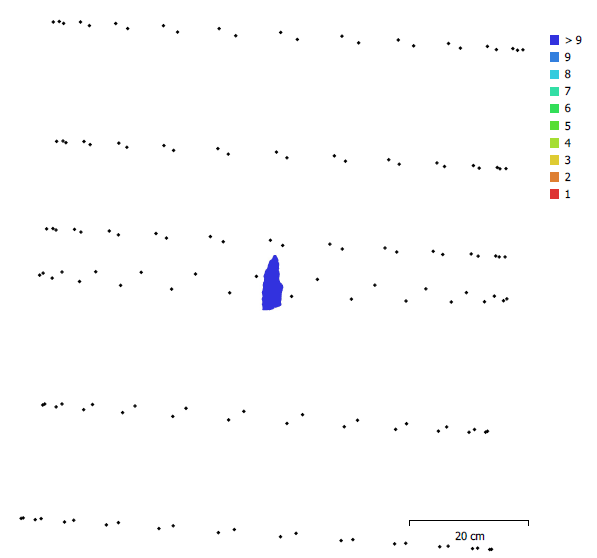


Fig. 2. Camera locations and image overlap of the 3D model of the bifacial piece TMBIII_UJ_A1_17.

5) Processing Parameters for the 3D model of the bifacial piece TMBIII_UJ_A1_16

**General**

Cameras 144

Aligned cameras 144

Markers 49

Scale bars 24

Coordinate system Local Coordinates (m)

Rotation angles Yaw, Pitch, Roll

**Point Cloud**

Points 270,647 of 356,434

RMS reprojection error 0.117386 (0.620041 pix)

Max reprojection error 0.377489 (22.0393 pix)

Mean key point size 4.09157 pix

Point colors 3 bands, uint8

Key points No

Average tie point multiplicity 2.7647

**Depth Maps**

Count 144

**Depth maps generation parameters**

Quality High

Filtering mode Mild

Processing time 21 minutes 32 seconds

**Dense Point Cloud**

Points 4,824,072

Point colors 3 bands, uint8

**Depth maps generation parameters**

Quality High

Filtering mode Mild

Processing time 21 minutes 32 seconds

**Dense cloud generation parameters**

Processing time 22 minutes 51 seconds

**Model**

Faces 1,500,000

Vertices 750,002

Vertex colors 3 bands, uint8

Texture 4,096 x 4,096, 4 bands, uint8

**Depth maps generation parameters**

Quality High

Filtering mode Mild

Processing time 21 minutes 32 seconds

**Reconstruction parameters**

Surface type Arbitrary

Source data Dense cloud

Interpolation Enabled

Strict volumetric masks Yes

Processing time 3 minutes 39 seconds

**Texturing parameters**

Mapping mode Adaptive orthophoto

Blending mode Mosaic

Texture size 4,096

Enable hole filling No

Enable ghosting filter No

UV mapping time 1 minutes 23 seconds

Blending time 50 seconds

**System**

Software name Agisoft Metashape Professional

Software version 1.6.2 build 10247

OS Windows 64 bit

RAM 63.85 GB

CPU Intel(R) Xeon(R) CPU E3-1505M v6 @ 3.00GHz

GPU(s) Quadro M2200

6) Proprieties of the 3D model of the bifacial piece TMBIII_UJ_A1_16

**Scaling**

Label Distance (m) Error (m)

target 1a_target 7a 0.0449075 -9.24726e-05

target 2a_target 8a 0.0449704 -2.95811e-05

target 3a_target 9a 0.0449154 -8.46143e-05

target 4a_target 10a 0.0448429 -0.000157058

target 5a_target 11a 0.0448095 -0.000190506

target 6a_target 12a 0.0448619 -0.000138099

target 13a_target 19a 0.0899791 -2.08946e-05

target 14a_target 20a 0.0899674 -3.25674e-05

target 15a_target 21a 0.0897844 -0.000215569

target 16a_target 22a 0.0898462 -0.000153761

target 17a_target 23a 0.0898021 -0.000197915

target 18a_target 24a 0.0899375 -6.2488e-05

target 1_target 7 0.0451131 0.000113129

target 2_target 8 0.0451761 0.000176091

target 3_target 9 0.0451121 0.000112142

target 4_target 10 0.0450302 3.01668e-05

target 5_target 11 0.0450202 2.01651e-05

target 6_target 12 0.0450546 5.45653e-05

target 14_target 20 0.0902015 0.000201529

target 15_target 21 0.0900685 6.8548e-05

target 16_target 22 0.0899277 -7.23403e-05

target 17_target 23 0.0900394 3.9362e-05

target 18_target 24 0.0902857 0.000285674

target 13_target 19 0.0902483 0.000248267

Total 0.000139541

**Resolution:** 0.0376 mm/pix

**Point density:** 706 points/mm²


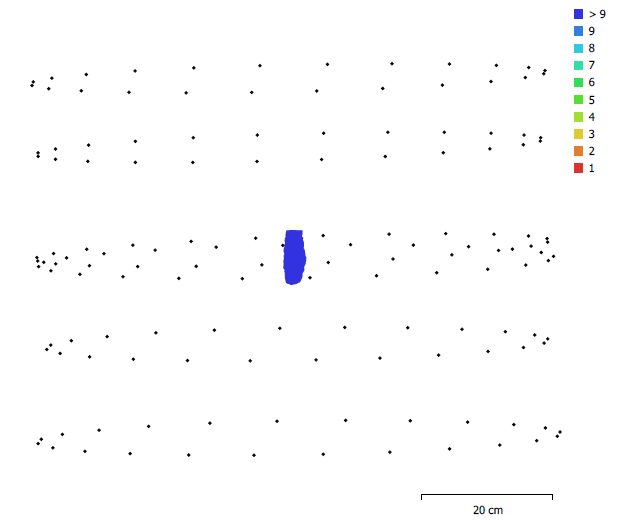


Fig. 3. Camera locations and image overlap of the 3D model of the bifacial piece TMBIII_UJ_A1_16.

**SI 4** Frequency distribution of lithic artefacts at Toumboura III. a) Frequency distribution of lithic artefacts > 20 mm and small debitage by *décapage* in square A1; b) Frequency distribution of lithic artefacts > 20 mm and small debitage by *décapage* in square B1

a)


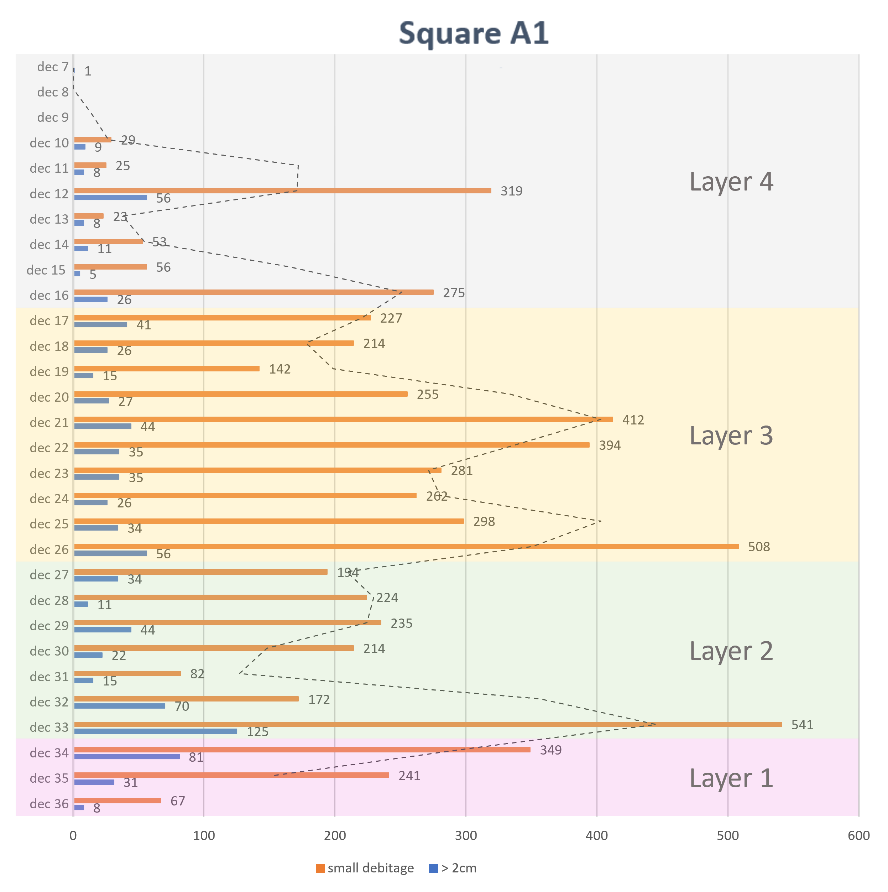


b)


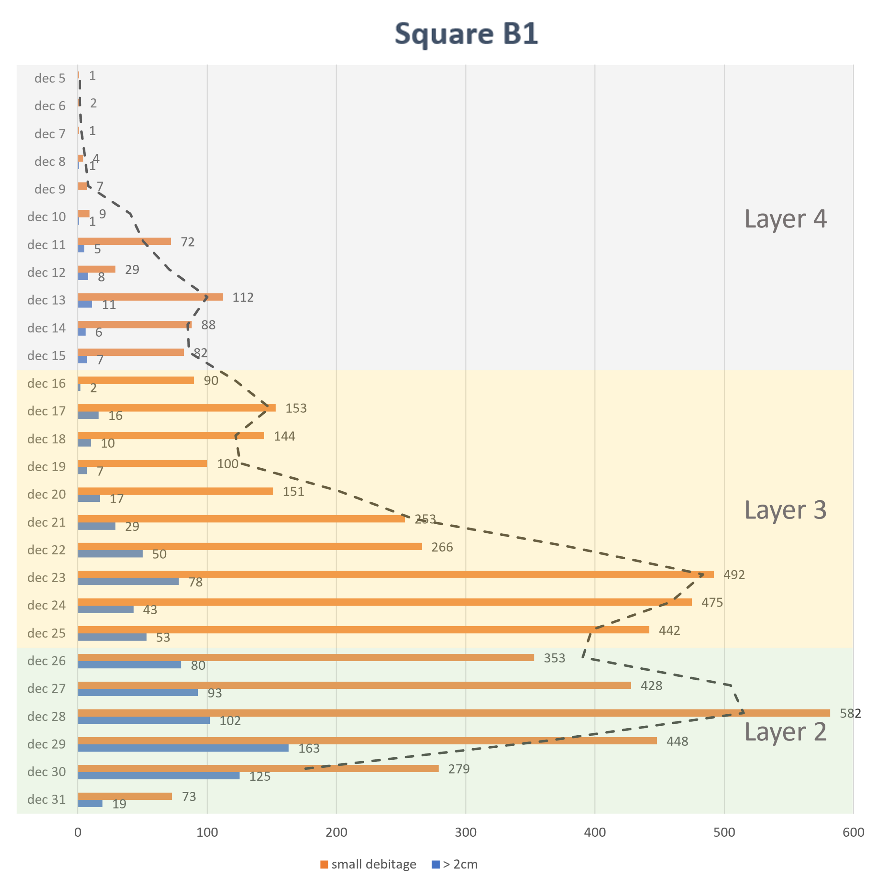


**SI 5** Descriptive statistics of length, width, thickness, weight, platform depth, External Platform Angle (EPA), and Internal Platform Angle (IPA) of the different phases of shaping flakes from Toumboura III

|  |  | **Early**  **shaping** |  | **Advanced**  **shaping** |  | **Final shaping** | **Re-**  **structuration** |
| --- | --- | --- | --- | --- | --- | --- | --- |
| Length (mm) | n | 71 |  | 92 |  | 8 | 3 |
|  | Mean | 30.3 | Mann–Whitney U test  (early/advanced): | 24.5 | Mann–Whitney U test  (advanced/final): | 18.9 | 21 |
|  | Median | 28.3 | **U = 2,447.5** | 22.5 | U = 218 | 19.5 | 21.5 |
|  | SD | 13.9 | **p = 0.0078** | 8.6 | p = 0.0555 | 2.3 | 2.3 |
|  |  |  |  |  |  |  |  |
| Width (mm) | n | 137 |  | 349 |  | 47 | 20 |
|  | Mean | 31.4 | Mann–Whitney U test (early/advanced): | 25.9 | Mann–Whitney U test (advanced/final): | 21.0 | 24.2 |
|  | Median | 29.2 | **U = 17,890** | 24.2 | **U = 4,848.5** | 21.0 | 23.5 |
|  | SD | 12.5 | **p < 0.0001** | 7.7 | **p < 0.0001** | 2.5 | 5.1 |
|  |  |  |  |  |  |  |  |
| Thickness (mm) | n | 277 |  | 683 |  | 77 | 32 |
|  | Mean | 8.5 | Mann–Whitney U test (early/advanced): | 5.6 | Mann–Whitney U test (advanced/final): | 4.3 | 5.6 |
|  | Median | 7.8 | **U = 50,055** | 5.0 | **U = 16,175** | 4.3 | 5.6 |
|  | SD | 4.1 | **p < 0.0001** | 2.2 | **p < 0.0001** | 0.8 | 1.5 |
|  |  |  |  |  |  |  |  |
| Weight (g) | n | 49 |  | 64 |  | 6 | 3 |
| *only completely preserved pieces considered* | Mean | 15.3 | Mann–Whitney U test (early/advanced): | 5.6 | Mann–Whitney U test (advanced/final): | 1.3 | 3.0 |
|  | Median | 7.0 | **U = 950.5** | 4.0 | **U = 58** | 1.0 | 2.0 |
|  | SD | 20.6 | **p = 0.0005** | 5.8 | **p = 0.0028** | 0.5 | 1.7 |
|  |  |  |  |  |  |  |  |
| Platform depth (mm) | n | 100 |  | 214 |  | 56 | 26 |
|  | Mean | 7.6 | Mann–Whitney U test (early/advanced): | 4.2 | Mann–Whitney U test (advanced/final): | 2.8 | 5.4 |
|  | Median | 6.6 | **U = 4,942** | 3.7 | **U = 3,517.5** | 2.6 | 5.1 |
|  | SD | 4.9 | **p < 0.0001** | 2.3 | **p < 0.0001** | 0.9 | 1.7 |
|  |  |  |  |  |  |  |  |
| Platform width (mm) | n | 67 |  | 161 |  | 45 | 19 |
|  | Mean | 20.1 | Mann–Whitney U test (early/advanced): | 13.6 | Mann–Whitney U test (advanced/final): | 10.0 | 18.7 |
|  | Median | 17.2 | **U = 3,017** | 12.5 | **U = 2,431** | 9.8 | 19.0 |
|  | SD | 10.4 | **p < 0.0001** | 6.3 | **p = 0.0006** | 3.7 | 4.4 |
|  |  |  |  |  |  |  |  |
| EPA (°) | n | 126 |  | 274 |  | 63 | 31 |
|  | Mean | 71.6 | Mann–Whitney U test (early/advanced): | 68.2 | Mann–Whitney U test (advanced/final): | 63.0 | 68.6 |
|  | Median | 74.0 | **U = 13,666** | 69.5 | **U = 5,480.5** | 62.0 | 70.0 |
|  | SD | 12.8 | **p = 0.0011** | 10.7 | **p < 0.0001** | 7.5 | 10.6 |
|  |  |  |  |  |  |  |  |
| IPA (°) | n | 126 |  | 274 |  | 63 | 31 |
|  | Mean | 110.1 | Mann–Whitney U test (early/advanced): | 110.8 | Mann–Whitney U test (advanced/final): | 112.5 | 106.8 |
|  | Median | 110.0 | U = 16,812 | 110.0 | U = 7,573.5 | 114.0 | 107.0 |
|  | SD | 10.5 | p = 0.6766 | 9.7 | p = 0.1262 | 7.4 | 13.0 |
